# *CsINV5*, a tea vacuolar invertase gene enhances cold tolerance in transgenic *Arabidopsis*

**DOI:** 10.1186/s12870-018-1456-5

**Published:** 2018-10-11

**Authors:** Wenjun Qian, Bin Xiao, Lu Wang, Xinyuan Hao, Chuan Yue, Hongli Cao, Yuchun Wang, Nana Li, Youben Yu, Jianming Zeng, Yajun Yang, Xinchao Wang

**Affiliations:** 1 National Center for Tea Plant Improvement, Tea Research Institute, Chinese Academy of Agricultural Sciences, Hangzhou, China; 2 Key Laboratory of Tea Biology and Resources Utilization, Ministry of Agriculture, Hangzhou, China; 3 College of Horticulture, Qingdao Agricultural University, Qingdao, Shandong China; 4 College of Horticulture, Northwest A & F University, Yangling, Shaanxi China; 5 Department of Tea Science, College of Horticulture, Fujian A & F University, Fuzhou, China

**Keywords:** *CsINV5*, Promoter, Cold tolerance, RNA-seq, *Arabidopsis thaliana*, *Camellia sinensis*

## Abstract

**Background:**

Vacuolar invertases (VINs) have been reported to regulate plant growth and development and respond to abiotic stresses such as drought and cold. With our best knowledge, the functions of VIN genes little have been reported in tea plant (*Camellia sinensis* L.). Therefore, it is necessary to develop research in this field.

**Results:**

Here, we identified a VIN gene, *CsINV5*, which was induced by cold acclimation and sugar treatments in the tea plant. Histochemical assays results showed that the 1154 bp 5′-flanking sequence of *CsINV5* drove β-glucuronidase (*GUS*) gene expression in roots, stems, leaves, flowers and siliques of transgenic *Arabidopsis* during different developmental stages. Moreover, promoter deletion analysis results revealed that an LTRE-related motif (CCGAAA) and a WBOXHVISO1 motif (TGACT) within the promoter region of *CsINV5* were the core *cis*-elements in response to low temperature and sugar signaling, respectively. In addition, overexpression of *CsINV5* in *Arabidopsis* promoted taproot and lateral root elongation through glucose-mediated effects on auxin signaling. Based on physiological and RNA-seq analysis, we found that overexpression of *CsINV5* improved cold tolerance in transgenic *Arabidopsis* mainly by increasing the contents of glucose and fructose, the corresponding ratio of hexose to sucrose, and the transcription of osmotic-stress-related genes (*P5CS1*, *P5CS2*, *AtLEA3*, *COR413-PM1* and *COR15B*) to adjust its osmotic potential.

**Conclusions:**

Comprehensive experimental results suggest that overexpression of *CsINV5* may enhance the cold tolerance of plant through the modification of cellular sugar compounds contents and osmotic regulation related pathways.

**Electronic supplementary material:**

The online version of this article (10.1186/s12870-018-1456-5) contains supplementary material, which is available to authorized users.

## Background

Sugars, such as sucrose (Suc), glucose (Glc) and fructose (Fru), are reported to act not only as osmoprotectants and ROS scavengers to stabilize membranes but also as signaling molecules to regulate gene expression when plants are exposed to low temperatures [1–4]. As a source of carbohydrate and energy, Suc could be hydrolyzed into monosaccharides by two types of enzyme exist in plants: Suc synthase (Sus, E.C. 2.4.1.13) and invertase (INV, E.C. 3.2.1.26). INVs irreversibly hydrolyze Suc into Glc and Fru, playing diverse roles in growth, development and response to abiotic and biotic stresses [5–8].

Based on optimum pH, INVs are classified into acid INVs (AI) and alkaline/neutral INVs (A/N-Inv). AIs are part of glycoside hydrolase family GH32 and are further subdivided as cell wall bound INVs (CWIN) and vacuolar INVs (VIN) according to their subcellular localization. AIs are β-fructofuranosidases, N-glycosylated proteins that contain a β-fructosidase motif (NDPD/AG) and a cysteine catalytic domain (WECP/VD). CWINs generate a Suc gradient essential for phloem unloading and carbon partitioning [9] and function in the development of plant tissues such as roots, flowers [10, 11], fruits [12] and seeds [13–15]. VINs are mainly involved in regulating cell expansion [16] and sugar composition in sugar-storing sink organs [17–19]. A/N-Invs are members of glycoside hydrolase family GH100 and mainly located in the cytoplasm, mitochondria or chloroplast [20, 21]. In addition, A/N-Invs are critical players in manipulating root and reproductive development [22–24].

Apart from their critical roles in plant growth and development, many studies have demonstrated that INVs respond to biotic and abiotic stresses in plants, though the molecular mechanism remains largely unknown [5, 25–28]. One possible mechanism of VIN responses to abiotic stress is changed activity following changed transcription, thus changing the ratio of hexose (Hex) to Suc to alter osmotic pressure in response to stimuli. For example, the induction by drought stress of a VIN gene, *Ivr2*, in the mature leaf, leaf sheath, and primary root of rice increases VIN activity, resulting in an accumulation of Hex that may participate in a signal transduction pathway or increase osmotic pressure to improve resistance to water deficits [29]. In contrast, the expression of *Ivr2* and VIN activity are reduced in young ovaries under drought stress, but the ratio of Hex to Suc still shows a close relationship with soluble INV activity, though in the ovary, sugars did not appear to contribute to osmotic acclimation [30]. In addition, Hex accumulation induced by VIN results in cold-induced sweetening in potato, whereas the suppression of VIN activity by transcription or post-translational regulation prevents this phenomenon [19, 26].

Tea plant is widely distributed in tropical and subtropical regions and represents one of the important economic crops in more than 20 countries. The growth and development of the tea plant are mainly limited by temperature, sunlight, water content, and soil fertility. Extremely low temperatures in winter and cold spells in late spring can seriously affect seasonal tea production and quality. As is well known, the freezing tolerance of plants could be induced by a period of low nonfreezing temperatures before the onset of winter, which is named as cold acclimation (CA). There are many changes occurred during CA process, involving gene expression, metabolism, and morphology [31]. In nature, CA is mainly trigged by low nonfreezing temperatures in late autumn or early winter [32], while it is induced by exposing plants to the low nonfreezing temperatures (2–6 °C) in the controlled environment chamber [33]. The freezing tolerance of acclimated plants is higher than that of nonacclimated plants. Nonacclimated *Arabidopsis*, for instance, had an EL50 (temperature resulted in 50% electrolyte leakage) of approximately − 4.5 °C, but the acclimated *Arabidopsis* had a lower EL50 values of approximately − 6 °C [34]. As previous study shown, with the average air temperature decreased to around 7 °C, the tea plants would undergo the CA process, and when the average air temperature increased to over 9 °C, tea plants would start the de-acclimation process. Moreover, a certain extent of cold acclimation can enhance the cold tolerance of tea plant [35]. Furthermore, there have many studies found the expression levels of many genes were changed in tea plants when they exposed to low nonfreezing temperatures (4 °C) or during natural CA process [36–40]. Recently, Wang et al. [41] revealed by RNA-seq analysis that the expression levels of many sugar-metabolizing genes were increased or reduced during CA, demonstrating that carbohydrate metabolism might be a major pathway in response to cold stress in the tea plant during CA. Afterwards, Yue et al. [42] further verified this hypothesis, finding that 59 sugar-related genes induced by low temperature, particularly a putative *INV* gene, *CsINV5*, which showed more than 100-fold higher expression during CA than before or after CA. The expression levels of *CsINV5* was also elevated at least 3-fold in leaves and 2-fold in roots of tea plant after 5 days of low nonfreezing temperature (4 °C) treatment as compared to normal cultivated tea plant [38], indicating that *CsINV5* may play an important role in cold stress response.

To better understand how *CsINV5* mediate the cold tolerance of tea plant, we analyzed the functions of 5′-flanking sequence of *CsINV5* drove β-glucuronidase (GUS) gene and the opening reading frame (ORF) of *CsINV5* overexpressed in transgenic *Arabidopsis*, respectively. In the present study, we demonstrate that a low-temperature-responsive element (CCGAAA) within the promoter of *CsINV5* is the core *cis*-element in cold stress response, and a sugar-related element (TGACT) is the main *cis*-element mediating sugar signaling. Furthermore, overexpression (OE) of *CsINV5* in *Arabidopsis* not only accelerated root growth through an osmotic-independent pathway but also enhanced cold resistance, mainly through the osmotic-dependent pathway.

## Results

### Phylogenetic analysis of CsINV5

**Fig. 1:**
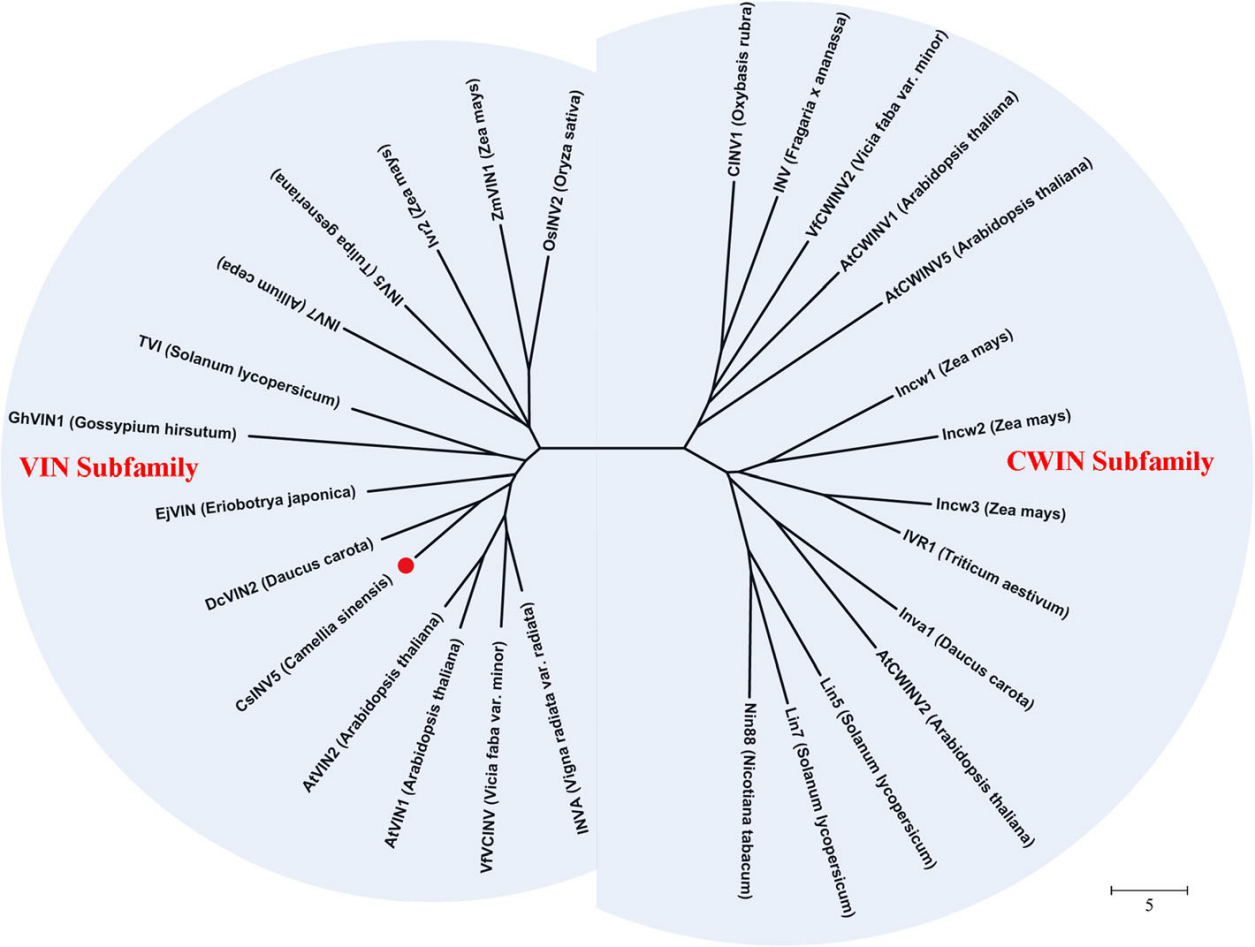
Phylogenetic analysis of CsINV5 and known SAIs. CsINV5 (KP053402, *Camellia sinensis*), GhVIN1 (ACQ82802, *Gossypium hirsutum*), INV5 (X97642, *Tulipa gesneriana*), INV7 (Y11230, *Allium cepa*), VfVCINV (Z49831, *Vicia faba var. minor*), DcVIN2 (Y18706, *Daucus carota*), EjVIN (KF938903, *Eriobotrya japonica*), AtVIN1 (At1g62660, *Arabidopsis thaliana*), AtVIN2 (At1g12240, *Arabidopsis thaliana*), Ivr2 (U31451, *Zea mays*), ZmVIN1 (U16123, *Zea mays*), OsINV2 (AF276703, *Oryza sativa*), TVI (NM_001247914, *Solanum lycopersicum*), INVA (D10265, *Vigna radiata var. radiata*), AtCWINV1 (At3G13790, *Arabidopsis thaliana*), AtCWINV2 (At3G52600, *Arabidopsis thaliana*), AtCWINV5 (At3G13784, *Arabidopsis thaliana*), CINV1 (X81792, *Oxybasis rubra*), INV (AF000520, *Fragaria x ananassa*), Lin7 (X91391, *Solanum lycopersicum*), Lin5 (X91389, *Solanum lycopersicum*), Nin88 (AF376773, *Nicotiana tabacum*), IVR1 (AF030420, *Triticum aestivum*), Incw1 (AF050129, *Zea mays*), Incw2 (AF050128, *Zea mays*), Incw3 (AF043346, *Zea mays*), Inva1 (X69321, *Daucus carota*), VfCWINV2 (Q43856, *Vicia faba var. minor*). CsINV5 is highlighted with a red dot

To investigate the evolutionary relationships of CsINV5, the amino acid sequence was aligned with those of known INVs, and a phylogenetic tree was constructed. As Fig. 1 shows, CsINV5 is a SAI that clusters into the VIN subfamily and has the closest relationship with DcVIN2 [43]; CsINV5 is also phylogenetically close to EjVIN1 and GhVIN1, the VINs of loquat [44] and cotton [45] respectively. In addition, the alignment result of the partial deduced protein sequences of VINs and CWINs indicated that 13 conserved regions reported from known SAIs, including four putative enzyme active site residues [46], the β-fructofuranosidase motif (NDPNG/A), and the RDP and WECP/VD motifs [47] were found in the protein sequence of CsINV5. Notably, more than 30 single amino acid differences were observed between CWINs and VINs (Additional file 1: Figure S1). In addition, we explored the genomic sequence of *CsINV5* using the tea plant genome described recently by Xia et al. [48]; the genomic DNA sequence of *CsINV5* contains 7 exons and 6 introns (Additional file 2: S1), and the second exon encodes a β-fructofuranosidase motif (DPN) reported as one of the smallest exons known in plants [49]. Based on the above results, we demonstrate that *CsINV5* encodes a VIN protein in tea plant.

### Expression of *CsINV5* is induced by CA and sugar treatment

**Fig. 2:**
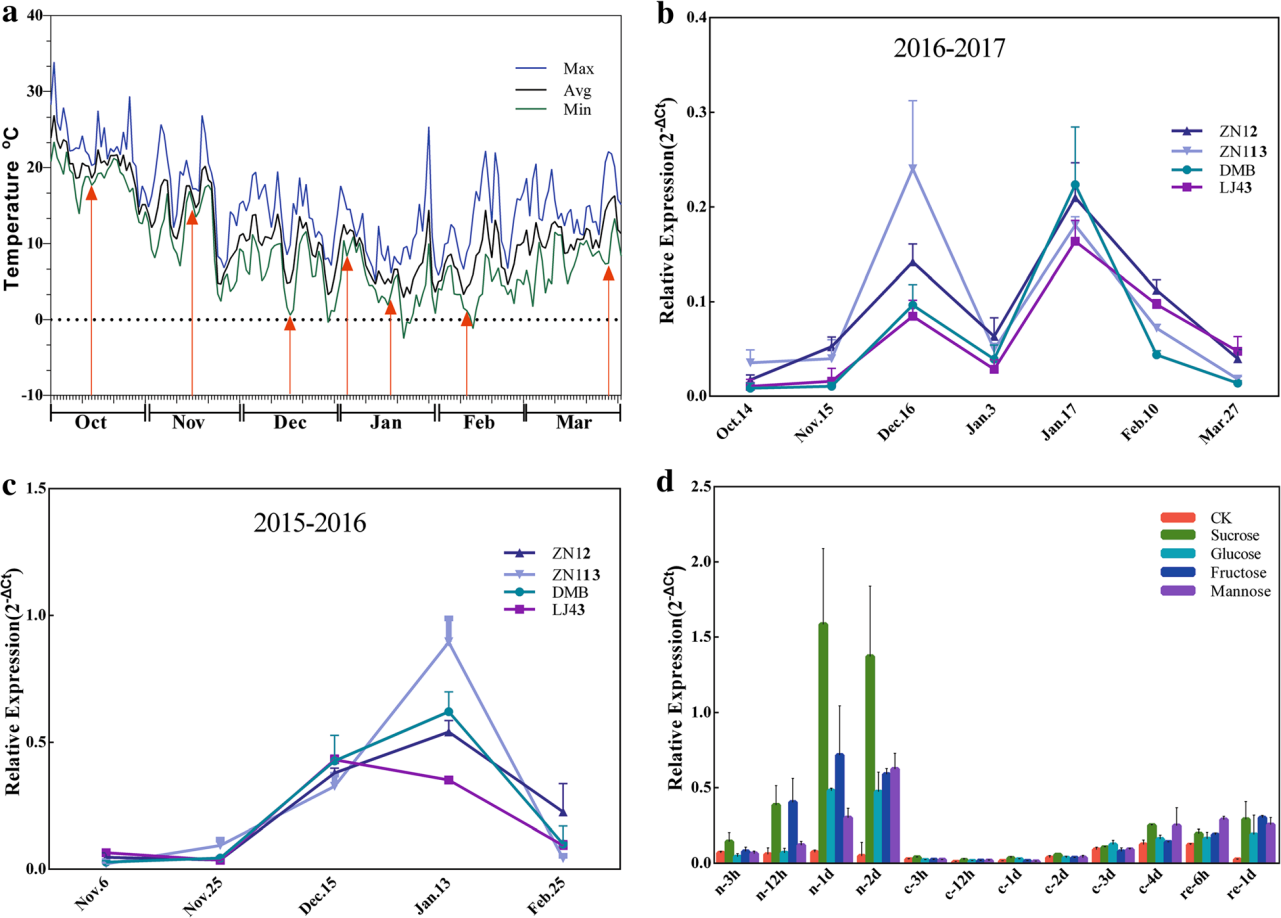
Expression analysis of *CsINV5* during CA periods in 2015–2016 and 2016–2017 and under different sugar and temperature conditions. **a** Changes in air temperature from October 2016 to March 2017. The maximum (Max), average (Avg) and minimum (Min) daily temperatures are shown. The red arrows indicate the Avg temperature of each sampling day. **b-c** Expression analysis of *CsINV5* in the mature leaves of four-tea cultivars during cold acclimation periods in 2015–2016 and 2016–2017 by using qRT-PCR. The expression pattern of *CsINV5* in different tea cultivars was showed with different color lines. **d** Expression analysis of *CsINV5* in the leaves of tea plant cultivar ‘LJ43’ under different sugar and temperature conditions by using qRT-PCR. ‘CK’ represents the sugar deficiency control, ‘n’ represents the normal temperature, ‘c’ represents the 4 °C treatment, ‘re’ represents the temperature recovered to normal level. All results were calculated by using the 2^–ΔCt^ method with *CsPTB* as housekeeping gene. Data are shown as the means ± SE (*n* = 3)

Previous study found the expression of *CsINV5* was induced at a high level during CA period (from Nov.2 to Dec.19), especially in Dec.19 that the expression level of *CsINV5* was elevated more than 300-fold, moreover, it was also induced at a high level during winter hardiness period (from Dec.19 to Jan.10) [42]. In the present study, we further compared the expression of *CsINV5* in four-tea cultivars during CA in 2015–2016 and 2016–2017. As Fig. 2b and c show, the expression of *CsINV5* was similar among different tea plant cultivars following temperature changes in winter. Briefly, *CsINV5* transcripts increased during CA with decreasing temperature (from late December to early January) and decreased during de-acclimation with increasing temperature (from late January to early February). In addition, we found exogenous sugars, including Suc, Glc and Fru, enhanced the expression of *CsINV5* in hydroponic seedlings of cultivar ‘LJ43’ in both normal and cold conditions (Fig. 2d), but the transcript levels of *CsINV5* were higher when treated with exogenous sugars under normal conditions.

### Promoter analysis of *CsINV5*

To decipher the functional mechanisms of the regulation of *CsINV5* expression, the 1154 bp sequence upstream of *CsINV5* was isolated from total genomic DNA of the ‘LJ43’ cultivar, and the *cis*-acting elements were predicted. The results showed that a TATA-box and a CAAT-box were located at − 65 bp and − 140 bp upstream of the translation start codon, respectively (Additional file 2: S1). Moreover, 18 motifs potentially involved in responding to biotic or abiotic stresses, light, hormones and sugars were found in this sequence (Table 1). Among these 18 motifs, 4 low-temperature-related *cis*-elements were predicted, including an LTR-related motif (CCGAAA, − 1078) and 3 MYC binding sites (CANNTG, − 453, − 369 and − 346) reported to interact with bHLH proteins at low temperatures [50]. In addition, a sugar-repressive element, SRE (TTATCCA, − 801), and a sugar-responsive element, WBOXHVISO1 (TGACT, − 124), were also found. These findings suggest that the promoter of *CsINV5* contains various motifs responding to exogenous and endogenous factors.

**Table 1:** Stress-, light-, hormone- and sugar-responsive elements in the 1154 bp 5′-flanking sequence of *CsINV5* as predicted by the PLantCARE website

Site name	Element	Sequence	Function	Copy	Position
Stresses	ARE	TGGTTT	Essential for the anaerobic induction	2	− 197, − 467
	HSE	AAAAAATTTC	Involved in heat stress responsiveness	2	−402, −551
	TC-rich repeats	ATTCTCTAAC	Involved in defense and stress responsiveness	1	− 337
	LTR	CCGAAA	Involved in low temperature responsiveness	1	− 1078
	MYC	CAACGTG/CACATG	Low temperature-related element	3	− 346, − 369, −453
Light	MRE	AACCTAA	MYB binding site involved in light responsiveness	1	−1054
	I-box:	GATAAGGGT	Light responsive element	1	− 741
	L-box	CTCACCTACCAA	Part of a light responsive element	1	− 491
	Sp1	CC(G/A)CCC	Light responsive element	2	− 170, −491
Hormones	ERE	ATTTCAAA	Ethylene-responsive element	1	− 399
	TCA-element	CAGAAAAGGA	Involved in salicylic acid responsiveness	1	− 142
Sugars	WBOXHVISO1	TGACT	SUSIBA2 bind to W-box element	1	−124
	SRE	TTATCCA	Alpha-amylase; MYB proteins; gibberellin; sugar starvation;	1	−801

#### Tissue-specific analysis

**Fig. 3:**
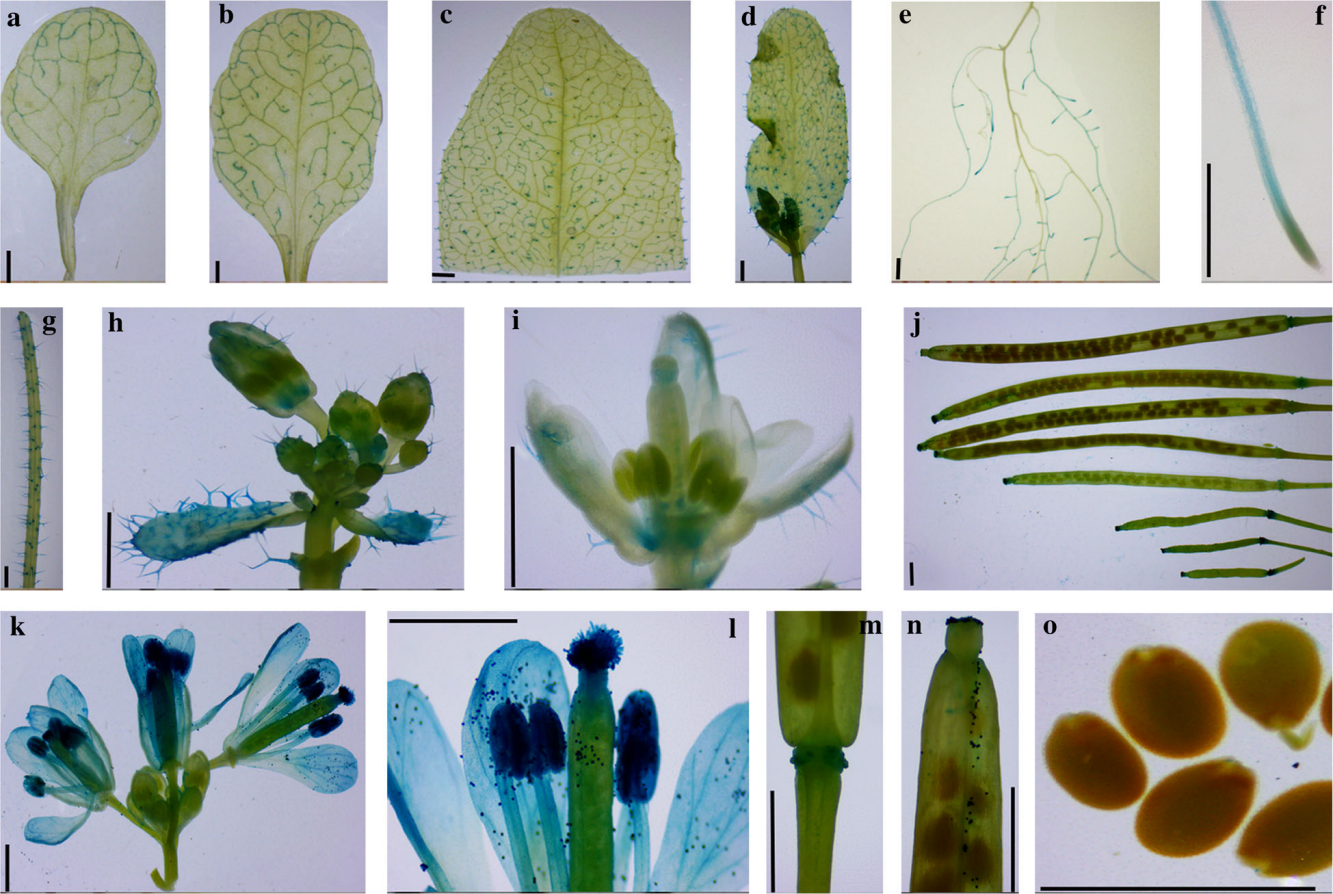
Histochemical GUS staining of transgenic *Arabidopsis* with *P1154CsINV5::GUS*. **a** partial first rosette leaf; **b** partial fourth rosette leaf; **c** mature rosette leaf; **d** cauline leaf; **e** partial root-zone; **f** root tip; **g** main stem; **h** raceme; **i** single bud; **j** siliques at different development stages; **k** flowers; **l** single flower; **m** peduncle; **n** silique apex; and **o** seeds. Scale bars = 1 mm

**Fig. 4:**
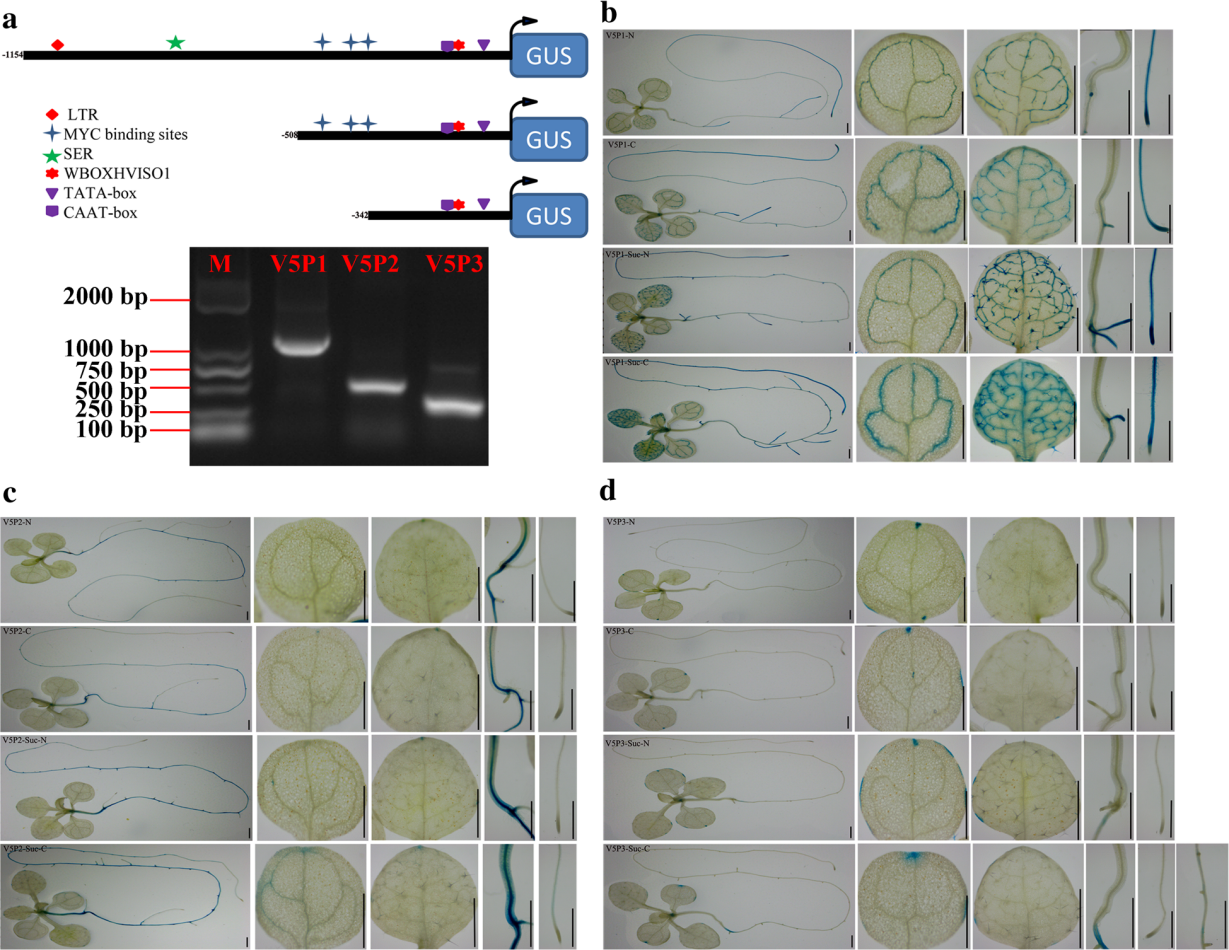
Promoter deletion analysis of *CsINV5* under Suc and different temperature exposure. **a** Schematic representation of chimeric *P1154CsINV5::GUS* constructs. Different symbols indicate different motifs of interest. Numbers represent distances from the translation start codon. **b-d** Histochemical GUS staining of transgenic *Arabidopsis* with *P1154CsINV5::GUS, P508CsINV5::GUS*, and *P342CsINV5::GUS* under normal-temperature and Sucrose-deleted conditions (V5P1-N, V5P2-N, and V5P3-N), low-temperature and Sucrose-deleted conditions (V5P1-C, V5P2-C, and V5P3-C), normal-temperature and Sucrose-added conditions (V5P1-Suc-N, V5P2-Suc-N, V5P3-Suc-N), and low-temperature and Sucrose-added conditions (V5P1-Suc-C, V5P2-Suc-C, V5P3-Suc-C), respectively. Scale bars = 1 mm

To verify the tissue specificity of *CsINV5*, three independent T_3_ homozygous transgenic lines that contain *P1154CsINV5::GUS* recombinant vector (Fig. 4a), were used for tissue-specific analysis. Histochemical staining results showed that the 5′-flanking sequence of *CsINV5* drove the transcription of the GUS reporter gene in various organs of transgenic *Arabidopsis* during different developmental stages (Fig. 3), though not in seeds. Moreover, we found that GUS activity in flowers increased with floral organ maturity, and the highest GUS activity was found in mature pollen. These results indicate that *CsINV5* is commonly expressed in various organs of the tea plant during different developmental stages and may play a role in modulating floral organ maturity.

#### Cold and sugar treatments

To explore the transcriptional regulatory mechanisms of *CsINV5* under cold or sugar conditions, we evaluated the responses to cold and sugar (3% Suc) of the proximal promoter region and its deletions (Fig. 4a). Briefly, the truncated promoter *P508CsINV5* (LTRE-related and SER motifs deleted) and *P342CsINV5* (LTRE-related, SER and MYC motifs deleted) were fused to the *GUS* coding sequence, and T_3_ homozygous transgenic *Arabidopsis* plants plus the *P1154CsINV5::GUS* transgenic plants were used for further analysis. Both WT and the empty vector (*pBI101::GUS*) transgenic *Arabidopsis* were regarded as control.

Histochemical assays revealed that the GUS staining in the leaves and roots of *P1154CsINV5::GUS* transgenic plants was significantly increased by cold and/or Suc treatment (Fig. 4b). The GUS staining in *P508CsINV5::GUS* transgenic plants planted on Suc-deleted medium showed no difference in either normal or cold conditions. However, when planted on Suc-added medium, the GUS activity in leaves, stems and roots of the *P508CsINV5::GUS* transgenic plants were all increased under normal conditions. Furthermore, *P508CsINV5::GUS* transgenic plants on Suc-added medium showed more GUS staining in stems and roots at low temperatures than at normal temperatures (Fig. 4c). A similar result was found in *P342CsINV5::GUS* transgenic plants (Fig. 4d). Besides, similar results were obtained in Glc or Fru treatments (Additional file 1: Figure S2), and control results are shown in Additional file 1: Figure S3a and b. Overall, we suggest that the LTRE-related motif, not the MYC binding sites, serves as the core *cis*-element regulating the transcriptional activity of *CsINV5* in response to cold and that the WBOXHVISO1 motif, as a sugar-responsive *cis*-acting motif, controls *CsINV5* transcription in response to both Suc signaling and cold stress.

### Overexpression analysis of *CsINV5* in transgenic *Arabidopsis*

#### Overexpressed *CsINV5* promotes root growth in transgenic *Arabidopsis*

**Fig. 5:**
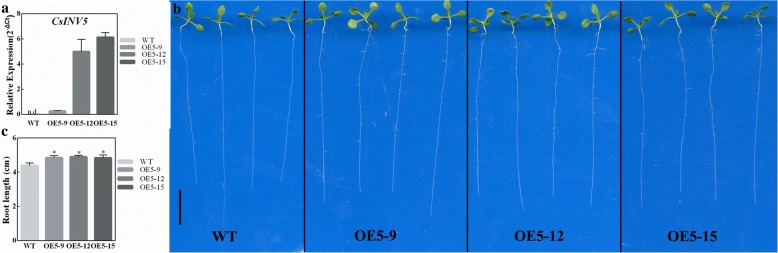
Expression of *CsINV5* in WT *Arabidopsis* promotes root growth. **a** The expression of *CsINV5* in the leaves of WT and *CsINV5*-OE plants. Data are shown as the means ± SE (*n* = 3). **b** Three *CsINV5*-OE lines showed a longer-root phenotype compared with WT. Scale bar = 10 mm. **c** The root lengths of three *CsINV5*-OE lines and WT *Arabidopsis* after 10 days of growth (each value represents the mean ± SE of 12 seedlings)

To investigate whether *CsINV5* mediates root growth, three homozygous transgenic overexpression lines (OE5–9, OE5–12 and OE5–15) with different transcript abundances were used in the following experiments (Fig. 5a). As Fig. 5b and c show, constitutively overexpressed *CsINV5* in *Arabidopsis* promoted taproot elongation in transgenic plants compared to WT plants; the root length was significantly longer in all investigated transgenic lines than in WT. Moreover, *CsINV5*-OE plants showed greater development of lateral roots (Fig. 5b).

#### Overexpression of *CsINV5* enhances cold tolerance in transgenic *Arabidopsis*

To explore how *CsINV5* participates in low-temperature response, *CsINV5*-OE (OE5–9, OE5–12 and OE5–15) and WT plants were exposed to 4 °C for 6 d, the temperature was reduced to − 1 °C for 2 d, and then leaves were collected to measure the Fv/Fm. Besides, the leaves exposed to − 6 °C for 8 h were collected to measure the EL.

**Fig. 6:**
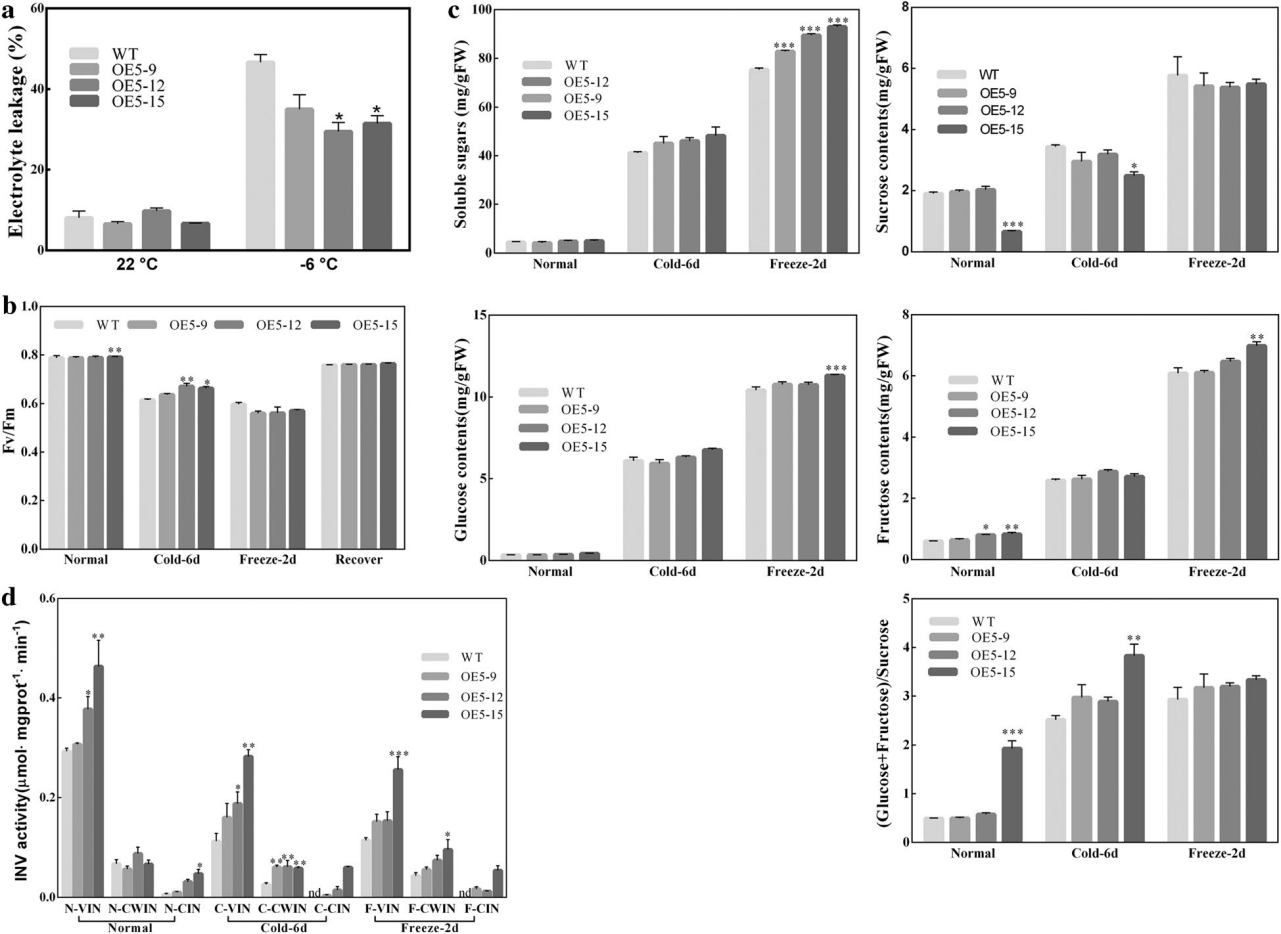
Analysis of EL, Fv/Fm, sugar contents and INV activities in *CsINV5*-OE plants and WT plants. **a** EL values in *CsINV5*-OE plants and WT plants under different temperature conditions. **b** Fv/Fm ratios in *CsINV5*-OE plants and WT plants under different temperature conditions. **c** Sugar contents, including TSS, Suc, Glc, Fru and the corresponding ratio of Hex/Suc, in *CsINV5*-OE and WT plants under different temperature conditions. **d** INV activities, including VIN, CWIN and CIN, in *CsINV5*-OE plants and WT plants under different conditions. Each experiment was performed with four replicates. Data are shown as the means ± SE (*n* = 4). Significant differences between *CsINV5*-OE and WT plants in the same conditions are indicated by one (*P* < 0.05) or two (*P* < 0.01) asterisks

Under normal conditions, the relative EL levels showed no significant difference between *CsINV5*-OE and WT plants. Under cold conditions, EL in *CsINV5*-OE was lower than in WT, and OE5–12 and OE5–15 showed significant differences compared to WT (Fig. 6a). Fv/Fm, a key parameter for photosynthetic status [51, 52], was higher in *CsINV5*-OE than in WT when plants were exposed to 4 °C for 6 d and significantly different in OE5–12 and OE5–15 compared to WT. Fv/Fm values were comparable in *CsINV5*-OE and WT plants exposed to − 1 °C for 2 d and recovered for 1 d (Fig. 6b).

On the other hand, all four types of sugar content (TSS, Suc, Glc and Fru) increased after cold treatment, and the contents of TSS, Glc, and Fru and the corresponding Hex/Suc ratio in *CsINV5*-OE were higher than in WT under chilling and freezing conditions (Fig. 6c); a significant difference existed in OE5–15 plants. In contrast, the Suc content in *CsINV5*-OE was slightly reduced compared to WT at low temperature. Among the INV activities, we found that VIN activity was significantly higher in *CsINV5*-OE than in WT under both normal and cold conditions, though the transcription of the gene (*AtVIN1*) corresponding to native VIN activity was reduced at low temperatures (Additional file 1: Figure S4a), which suggests that *CsINV5* was successfully expressed in transgenic *Arabidopsis*. The activity of CWIN in *CsINV5*-OE was not significantly affected but slightly lower in WT plants, and CIN activity was not detected in WT plants under cold conditions. These results indicate that *CsINV5*-OE was associated with higher INV activity and increased the Hex/Suc ratio by hydrolyzing much more Suc into Glc and Fru. Based on these data, *CsINV5* overexpression in *Arabidopsis* enhanced cold tolerance.

#### Overexpression of *CsINV5* influenced the transcription of cold-related genes

To explore the molecular mechanisms underlying the *CsINV5*-mediated increase in cold tolerance in transgenic *Arabidopsis*, the leaves of WT and *CsINV5*-OE plants (OE5–15) under normal and cold conditions were subjected to RNA-seq analysis. After quality-based trimming, 83.59 Gb of clean data was obtained from 12 RNA-seq libraries, and the Q30 of each sample was not less than 90.65% (Additional file 3: Table S1; Submission number in NCBI: SRR6057439-SRR6057450). Twelve transcripts were selected to validate the RNA-seq data by qRT-PCR, and Additional file 1: Figure S5 shows that all had similar expression patterns between RNA-seq and qRT-PCR, suggesting that the RNA-seq data are reliable.

**Fig. 7:**
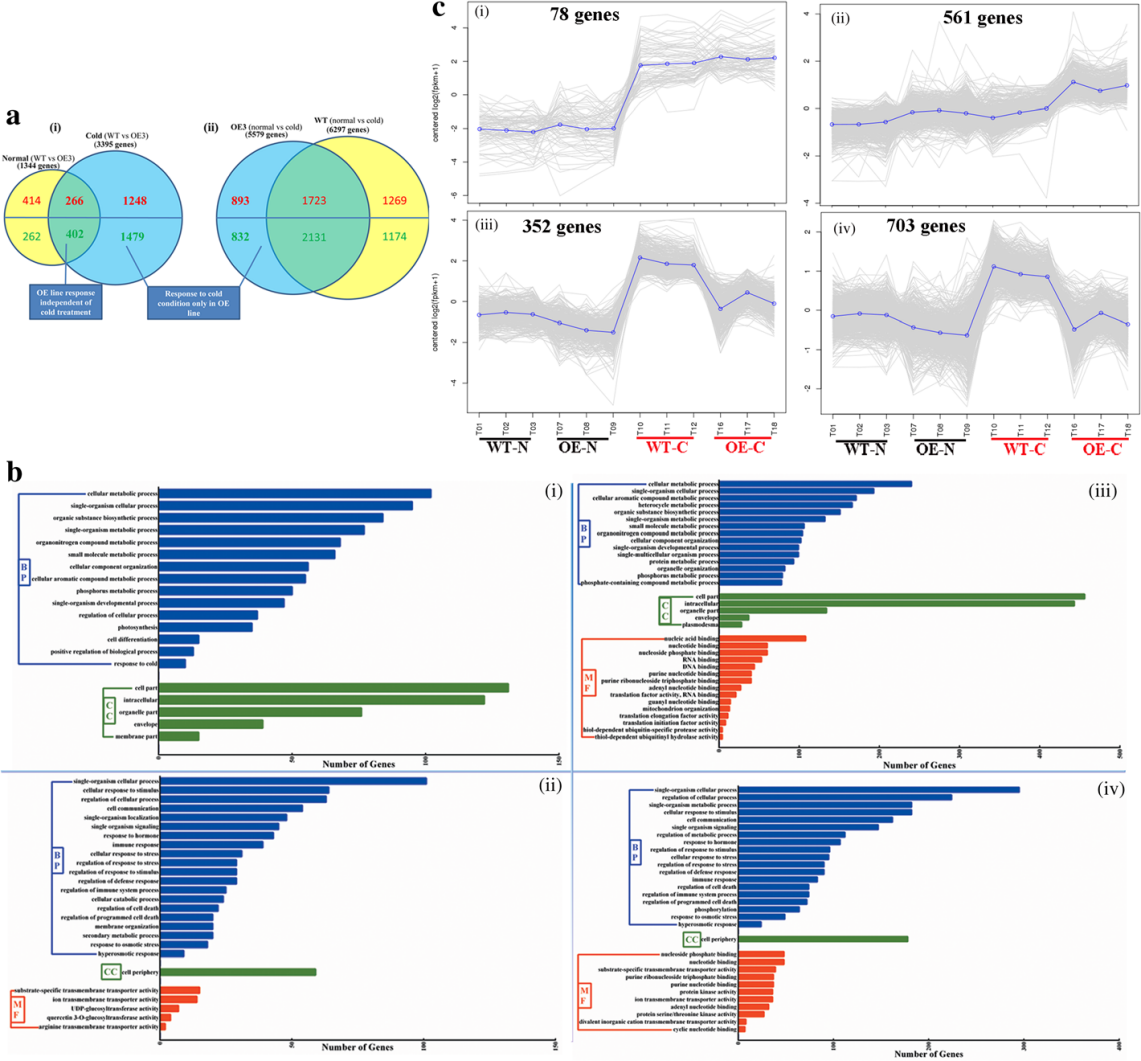
RNA-seq analysis of *CsINV5-*OE and WT plants. **a** Venn diagrams showing the numbers of DEGs (*FDR* < 0.05, log_2_FC) in *CsINV5-*OE plants compared to WT plants under normal and cold conditions (WT vs OE) and the DEGs (*FDR* < 0.05, log_2_FC) in *CsINV5-*OE plants and WT plants under normal and cold conditions. **b** Partial GO terms enriched in the overlap area (266 up-regulated genes (i) and 402 down-regulated genes (ii) and specifically regulated area (1248 up-regulated genes (iii) and 1479 down-regulated genes (iv) between OE and WT plants in normal and cold conditions, respectively (*FDR* < 0.05). **c** Partial co-expression patterns of DEGs between OE and WT plants in normal and cold conditions (*FDR* < 0.05, K-means = 10). (i-ii) DEGs up-regulated in *CsINV5-*OE plants compared to WT plants; (iii and iv) DEGs down-regulated in *CsINV5-*OE plants compared to WT plants

DEGs between WT and *CsINV5*-OE plants under normal or cold conditions were selected based on fold change (FC) ≥ 2 and *FDR* < 0.05. Under normal conditions, 1344 DEGs were obtained from WT and OE plants, while nearly three times as many DEGs (3395) were found between WT and *CsINV5*-OE plants under cold conditions, implying that *CsINV5* played a major role in cold stress response in transgenic *Arabidopsis* (Fig. 7ai). Moreover, 5579 DEGs containing 893 up-regulated genes and 832 down-regulated genes were specifically differentially expressed in *CsINV5*-OE plants, but the other DEGs were commonly differentially expressed both in WT and *CsINV5*-OE plants under cold condition. However, 6297 DEGs were found in WT plants under cold conditions (Fig. 7aii), indicating that metabolic changes in WT were more extensive at low temperature.

In *CsINV5*-OE plants, 668 DEGs (266 up-regulated and 402 down-regulated) were independent of temperature (Fig. 7ai). GO enrichment analysis, including biological process, cellular component and molecular function, was performed with an *FDR* < 0.05. For biological process, the GO terms enriched among the 266 up-regulated genes were mainly ‘photosynthesis’, ‘cell differentiation’ and ‘response to cold’, while the 402 down-regulated genes were mainly involved in responses to abiotic and biotic stimuli, defense and immunity (partial GO terms are listed in Fig. 7bi and ii and Additional file 4: Tables S2 and S3). Similarly, the 1248 up-regulated genes and 1479 down-regulated genes in *CsINV5*-OE plants under cold conditions showed similar GO enrichment results compared to above GO terms, respectively. For biological process, the 1248 specific up-regulated DEGs were enriched in ‘nucleic acid binding’, ‘RNA binding’, ‘DNA binding’ and ‘reactive oxygen species’, while the 1479 down-regulated DEGs were mainly involved in responses to abiotic and biotic stimuli, defense and immunity (partial GO terms are listed in Fig. 7biii and iv and Additional file 4: Tables S4 and S5).

Common DEG expression patterns were evaluated with K-means = 10 (Fig. 7c and Additional file 1: Figure S6). Six hundred and thirty-nine up-regulated DEGs clustered into two expression patterns with higher expression in *CsINV5*-OE than in WT under cold conditions (Fig. 7ci and ii). These genes included many cold-related genes (*CPN60B*, *CP29*, *RPL23AB*, etc.), osmotic-stress-related genes (*ATLEA3*, *COR413-PM1*, *COR15B*, *P5CS1*, *P5CS2*, etc.), and water-deprivation-related genes (*CER1, CR88*, etc.), which were all specifically up-regulated in *CsINV5*-OE (Table 2). In addition, 1055 genes that clustered as down-regulated DEGs were highly induced in WT but unchanged or slightly induced in *CsINV5*-OE at low temperatures (Fig. 7ciii and iv). Among these were many genes that respond to cold (*CBF1*, *AtCDPK1*, *AtFER1*, etc.), osmotic stress (*AtMYB50*, *MYB108*, *HSP17.6II*, *AtSWEET15*, etc.), and water deprivation (*NFYA5*, *DREB19*, *AtATG18F*, etc.), which were all specifically down-regulated in *CsINV5*-OE compared to WT (Table 2).

**Table 2:** RNA-seq analysis of WT plants and *CsINV5*-OE plants

ID	Gene name	Description	log_2_FC			
			WT vs OE		Normal vs Cold	
			Normal	Cold	WT	OE
Specific up-regulated genes						
Response to cold						
AT1G20020	*ATLFNR2*	NADP (H) oxidoreductase	normal	1.03	normal	normal
AT1G55490	*CPN60B*	Chaperonin 60 beta	normal	1.94	1.03	2.01
AT1G63940	*MDAR6*	Monodehydroascorbate reductase 6	normal	1.26	normal	normal
AT1G67090	*RBCS1A*	Rubisco small subunit (RBCS) multigene family	normal	1.37	normal	normal
AT2G35040	*AICARFT*	IMPCHase bienzyme family protein	normal	1.09	normal	normal
AT2G37190	*RPL12A*	Ribosomal protein L11 family protein	normal	1.02	normal	normal
AT2G37220	*–*	Encodes a chloroplast RNA binding protein.	normal	1.70	normal	normal
AT3G08000	*–*	RNA-binding (RRM/RBD/RNP motifs) family protein	normal	1.13	normal	normal
AT3G13470	*CPN60B2*	Chaperonin-60 beta2	normal	2.45	normal	2.10
AT3G23700	*SRRP1*	S1 RNA-binding ribosomal protein 1	normal	1.59	normal	normal
AT3G49910	*RPL26A*	Translation protein SH3-like family protein	normal	1.04	normal	normal
AT3G53460	*CP29*	Chloroplast RNA-binding protein 29	normal	2.34	normal	1.01
AT3G54050	*HCEF1*	Chloroplastic fructose 1, 6-bisphosphate phosphatase.	normal	1.65	normal	normal
AT3G55280	*RPL23AB*	Ribosomal protein L23A	normal	1.00	normal	1.13
AT4G04330	*–*	Homologue of cyanobacterial RBCX 1	normal	1.22	normal	normal
AT4G24280	*cpHsc70–1*	Chloroplast heat shock protein 70–1	normal	1.47	normal	1.31
AT5G20720	*CPN20*	Chaperonin 20	normal	1.71	normal	1.21
AT5G50250	*CP31B*	Chloroplast RNA-binding protein 31B	normal	1.17	normal	1.04
AT5G54770	*THI1*	Thiazole requiring	normal	1.12	normal	normal
Response to osmotic						
AT1G02820	*ATLEA3*	Late embryogenesis abundant 3 (LEA3) family protein	normal	1.40	4.77	4.95
AT1G73570	*–*	HCP-like superfamily protein	normal	1.53	normal	1.31
AT2G15970	*COR413-PM1*	Cold regulated 413 plasma memberane 1	normal	1.13	3.58	4.06
AT2G38230	*ATPDX1.1*	Pyrodoxine biosynthesis 1.1	normal	1.44	normal	normal
AT2G39800	*P5CS1*	Delta1-pyrroline-5-carboxylate synthase 1	normal	1.46	normal	1.83
AT2G42530	*COR15B*	Cold regulated 15B	normal	2.03	6.50	7.60
AT3G04770	*RPSAb*	40s ribosomal protein SA B	normal	1.14	normal	normal
AT3G14940	*ATPPC3*	Cytosolic phosphoenolpyruvate carboxylase 3	normal	1.64	1.03	1.16
AT3G55610	*P5CS2*	Delta1-pyrroline-5-carboxylate synthase 2	normal	1.57	normal	2.47
AT5G01410	*PDX1*	Aldolase-type TIM barrel family protein	normal	1.09	normal	1.22
AT5G44110	*ATPOP1*	*Arabidopsis thaliana* non-intrinsic ABC protein	normal	1.05	4.91	5.25
Response to water deprivation						
AT1G02205	*CER1*	Fatty acid hydroxylase superfamily	normal	1.12	7.08	4.89
AT2G04030	*CR88*	Heat shock protein 88	normal	1.81	normal	1.80
AT3G08000	*–*	RNA-binding (RRM/RBD/RNP motifs) family protein	normal	1.13	normal	normal
Specific down-regulated genes						
Response to cold						
AT1G01560	*ATMPK11*	Member of MAP Kinase family	normal	−1.11	1.19	normal
AT1G18890	*ATCDPK1*	Calcium-dependent protein kinase 1	normal	−1.17	2.02	1.48
AT1G29690	*CAD1*	Constitutively activated cell death 1	normal	−1.82	2.12	normal
AT1G51660	*ATMKK4*	Mitogen-activated protein kinase kinase 4	normal	−1.12	normal	normal
AT1G52890	*ANAC019*	NAC domain containing protein19	normal	−2.73	1.98	normal
AT1G66400	*CML23*	Calmodulin-like protein	normal	−2.24	2.12	normal
AT2G04430	*atnudt5*	Nudix hydrolase homolog 5	normal	−1.81	2.24	normal
AT2G04450	*ATNUDT6*	Nucleoside diphosphates linked to some moiety X 6	normal	−1.74	3.83	2.12
AT2G17290	*CPK6*	Calcium-dependent protein kinase family protein	normal	−1.28	1.13	normal
AT2G22300	*CAMTA3*	Calcium-binding transcription activator 3	normal	−1.04	normal	normal
AT2G30250	*WRKY25*	WRKY DNA-binding protein	normal	−1.59	1.17	normal
AT2G38470	*WRKY33*		normal	−2.53	2.29	normal
AT2G40140	*CZF1*	Salt-inducible zinc finger 2	normal	−1.38	2.93	2.30
AT3G03050	*CSLD3*	Cellulose synthase like D3	normal	−1.02	1.04	normal
AT3G05360	*AtRLP30*	Receptor like protein 30	normal	−1.84	2.77	1.86
AT3G11820	*SYP121*	Syntaxin related protein 1	normal	−1.42	1.28	normal
AT3G49530	*ANAC062*	NAC domain containing protein 62	normal	−1.70	2.41	1.03
AT3G52400	*SYP122*	Syntaxin of plants 122	normal	−2.56	2.19	normal
AT4G02330	*ATPMEPCRB*	Pectin methylesterase 41	normal	−1.63	2.54	1.27
AT4G08500	*MEKK1*	MAPK/ERK Kinase Kinase 1	normal	−1.33	1.22	normal
AT4G25490	*CBF1*	C-repeat/DRE binding factor 1	normal	−1.26	4.98	3.72
AT4G26120	*NPR2*	Ankyrin repeat family protein	normal	−2.03	1.99	normal
AT5G01600	*ATFER1*	*Arabidopsis thaliana* ferretin 1	normal	−1.45	1.84	1.03
AT5G01820	*ATSR1*	Serine/Threonine protein Kinase 1	normal	−1.16	1.47	normal
AT5G02490	*MED37D*	Heat shock protein 70 (Hsp 70) family protein	normal	−2.12	4.70	3.01
AT5G13320	*PBS3*	AVRPPHB susceptible 3	normal	−3.20	3.67	normal
AT5G16910	*ATCSLD2*	Cellulose-synthase like D2	normal	−1.40	1.61	1.08
AT5G26920	*CBP60G*	CAM-binding protein 60-like G	normal	−3.69	3.62	normal
AT5G42050	*NRP*	Asparagine-rich protein	normal	−1.33	1.58	normal
AT5G57560	*TCH4*	Xyloglucan endotransglucosylase/hydrolase 22	normal	−2.72	2.65	1.22
AT5G58670	*ATPLC1*	*Arabidopsis thaliana* phospholipase C	normal	−1.04	normal	normal
AT5G67340	*PUB2*	ARM repeat superfamily protein	normal	−2.32	3.82	2.01
Response to osmotic						
AT1G15520	*PDR12*	*Arabidopsis thaliana* ATP-binding cassette G40	normal	−3.13	2.13	normal
AT1G57560	*AtMYB50*	MYB domain protein	nd	−2.90	2.63	normal
AT3G06490	*MYB108*		normal	−2.81	1.42	normal
AT3G10500	*anac053*	NAC domain containing protein 53	normal	−1.38	normal	normal
AT3G14050	*RSH2*	RELA/SPOT homolog 2	normal	−2.10	2.21	1.04
AT3G22160	*JAV1*	Jasmonate-associated VQ motif gene 1	normal	−1.19	1.04	normal
AT3G28210	*PMZ*	Encodes a putative zinc finger protein	normal	−2.91	3.00	normal
AT4G15120	*–*	VQ motif-containing protein	normal	−2.57	3.07	normal
AT4G34390	*XLG2*	Extra-large GTP-binding protein 2	normal	−2.27	1.80	normal
AT4G36990	*HSF4*	Haliana heat shock factor 4	normal	−1.19	0.51	normal
AT5G13170	*SAG29*	Senescense-associated gene29	normal	−3.53	6.25	3.12
AT5G39720	*AIG2L*	Avirulence induced protein 2 like protein	nd	−2.90	2.82	normal
Response to water deprivation						
AT1G08920	*ESL1*	Early response to dehydration SIX-like 1	normal	−1.76	3.42	2.14
AT1G02930	*GSTF6*	*Arabidopsis thaliana* gluatione s-transferase F3	normal	−1.54	normal	normal
AT1G32870	*ANAC13*	NAC domain containing protein 13	normal	−1.12	2.56	1.74
AT1G32940	*ATSBT3.5*	Subtilase family protein	normal	−1.82	2.37	1.42
AT1G32950	*–*		normal	−3.30	4.69	1.15
AT1G54160	*NFYA5*	Nuclear factor Y A5	normal	−1.29	1.80	1.20
AT2G38340	*DREB19*	Dehyration response element-binding protein 19	normal	−2.62	2.20	normal
AT2G45570	*CYP76C2*	Cytochrome P450	normal	−2.14	2.71	1.41
AT3G56880	*–*	VQ motif-containing protein	normal	−1.33	1.80	normal
AT4G12250	*GAE5*	UDP-D-glucuronate 4-epimerase	normal	−1.26	1.50	normal
AT5G54730	*ATATG18F*	Yeast autophagy 18 F-like protein	normal	−1.04	1.26	normal

Differentially expressed genes (*FDR* < 0.05, log_2_FC) between WT plants and *CsINV5*-OE plants under cold conditions were annotated with the GO terms ‘response to cold’, ‘response to osmotic stress’ and ‘response to water deprivation’. The log_2_FC values between WT plants and *CsINV5*-OE plants under normal conditions, and log_2_FC values in *CsINV5*-OE plants or WT plants between normal and cold condition are also shown

“—” indicates the gene has not been named; “nd” represents that the FC value was not detected in DEGs expression analysis results. “normal” indicates the gene was not differentially expressed between these two conditions. The negative value represents the gene was down-regulated, and the positive value represents the gene was up-regulated

In summary, based on DEG expression patterns and functional annotations, we found that many cold-induced genes involved in the CDPK, MAPK, CBF/COR and ABA-dependent or -independent pathways had unchanged or slightly increased expression levels in *CsINV5*-OE plants exposed to cold stress. However, some genes encoding RNA-binding and chaperonin proteins and some osmotic-stress-related genes had higher expression levels in *CsINV5*-OE plants exposed to cold stress. Therefore, combined with the values of EL, Fv/Fm and sugar contents and the transcription levels of many osmotic-stress-related genes between WT and *CsINV5*-OE plants under low temperature, we demonstrate that the tolerance of *CsINV5*-OE to low temperature was stronger than that of WT.

## Discussion

### *CsINV5* encodes a VIN protein involved in cold stress response

Both chilling (< 20 °C) and freezing (< 0 °C) temperatures are belong to cold stress, which detrimentally affects the growth and development of plants and seriously limits the spatial distribution of plants and crop yields [53]. Most temperate plants acquire more freezing tolerance by exposing to non-freezing low temperatures (mainly range 2 to 6 °C in the controlled environment chamber), a process known as CA. Numerous of physiological, biochemical and molecular are changed during CA, including the remodeling of cell and tissue structures, the reprogramming of metabolism and gene expression [32]. In tea plant, previous studies showed that the expression level of *CsINV5* was increased 3 times in ‘LJ43’ cultivar when they exposed to a non-freezing low temperatures (4 °C) for 5 days as compared to the normal cultivated ‘LJ43’ cultivar [38]. In addition, the expression of *CsINV5* was also significantly induced by natural CA process [42]. In the present study, a similar expression patterns were also found in four-tea cultivars (DMB, LJ43, ZN12, and ZN113) during CA (Fig. 2b and c), which suggest *CsINV5* may function similarly in cold stress response among different tea plant cultivars, but the mechanism by which this gene participates in cold stress response remains unknown. In the present study, we found that *CsINV5* encodes a soluble protein with a molecular mass of nearly 80 kDa (Additional file 1: Figure S7). The protein properties of this gene are similar to those of known VIN proteins [47, 54, 55] (Fig. 1 and Additional file 1: Figure S1), suggesting that *CsINV5* encodes a functional VIN protein.

### The promoter region of *CsINV5* contains core motifs that respond to cold stress, sugar signaling and reproductive growth

The promoter region contains multiple *cis*-elements that can bind to various types of transcription factors to mediate the expression of downstream genes. By using deletion analysis, electrophoretic mobility shift analysis (EMSA) and synthetic oligonucleotides, Dunn et al. [56] identified an LTRE-related motif, CCGAAA, as the binding site of a low-mobility nuclear protein complex in the 42 bp low-temperature-responsive promoter region of *blt4.9*. Furthermore, they found the low-temperature responsiveness to basal levels would be decreased as this motif mutated, which suggests that CCGAAA is an alternative LTRE participating in low-temperature response. In the present study, we found that the same LTRE-related motif (CCGAAA) was proven to respond to cold stress based on our promoter deletion analysis in transgenic *Arabidopsis*, suggesting that the LTRE-related motif within the *CsINV5* promoter may serve as the core binding site of some low-mobility nuclear protein to positively regulate the expression of *CsINV5* under cold conditions.

On the other hand, many reports have shown that *INV* gene transcription is regulated by exogenous sugar treatment [44, 57–59]. McLaughlin and Boyer [60] reported that Suc feeding rescued the transcription of a VIN gene, *ZmIVR2*, which was reduced by water deficits. In addition, the results of promoter analyses have revealed the role of *INV* gene in response to sugar treatment [61–63]. Ou et al. [64] demonstrated that the promoter region from − 118 to − 551 bp of a VIN gene, *StvacINV1*, contained seven SREs (TTATCC) motifs, where they found this promoter region was required for negative response to Suc/Glc. Similarly, Rabot et al. [63] demonstrated that the − 595 to − 468 bp promoter region of *RhVI1*, which contains an SRE, was required for negative response to Suc and Fru in the dark. Moreover, the − 468 to − 307 bp promoter region of *RhVI1*, which also contains a WBOXHVISO1 (TGACT), promoted a significant induction of GUS activity by Suc and Fru in the dark, compared with mannitol treatment. SREs are recognized by three MYB transcription factors that induce the expression of α-amylase under Suc and Glc starvation in rice [65]. But the WBOXHVISO1 motif is recognized by SUSIBA2, a member of the WRKY family, which positively regulates the transcription of isoamylase in response to Suc during barley seed development [66]. In agreement with these findings, we found that the expression of *CsINV5* was induced by sugar treatments at normal or low temperatures (Fig. 2d). In addition, the results of the promoter deletion analysis further confirmed this phenomenon (Fig. 4), which suggesting that the sugar-responsive element (WBOXHVISO1) but not the SRE within the promoter region of *CsINV5* may act as the core motif to bind to a putative WRKY transcription factor to induce the transcription of *CsINV5* in response to sugar and/or cold treatments.

In addition to response to environmental stimuli and sugar treatment, VINs also play an important role in plant reproductive growth. Direct evidence was provided by Wang and Ruan [16]; silencing a VIN gene, *GhVIN1*, in cotton reproductive organs reduced viable seed production due to pollination failure and impaired male and female fertility. In our study, the promoter of *CsINV5* showed the highest GUS activity in mature pollen of transgenic *Arabidopsis*, and this result is consistent with the tissue-specific expression pattern of *CsINV5* in the tea plant, where we previously found the transcription abundance of *CsINV5* in flowers of ‘LJ43’ cultivar is nearly 90-fold than that in roots [38]. Thus, *CsINV5* may exert strong control over reproduction from male and female fertility to floral development, but the mechanism remains to be explored.

### *CsINV5* overexpression in *Arabidopsis* promotes root elongation through an osmotic-independent pathway

Many studies have reported that VINs play an important role in regulating plant cell expansion and cell division [45, 67–69]. A similar result was obtained in our study, where the overexpression of *CsINV5* enhanced both taproot and lateral root elongation in transgenic *Arabidopsis*, indicating that *CsINV5* regulates cell expansion and cell division. However, the mechanism by which VINs regulate root elongation is not yet clear, and different mechanisms of VIN-regulated root elongation may exist in different species. As Wang et al. [45] demonstrated a VIN gene, *GhVIN1*, regulates the elongation of cotton fibers and the roots of transgenic *Arabidopsis* in osmotic-dependent and -independent manners, respectively, based on the relative contributions of sugars to sap osmolality. In *Arabidopsis* roots, the combined Hex and Suc concentrations are extremely low, which indicates that their osmotic contribution is negligible. Therefore, we suggest that *CsINV5* increased root elongation in transgenic *Arabidopsis* independently of the osmotic pathway. In addition, Wang et al. [70] found that *GhVIN1* may mediate Hex signaling to regulate fiber initiation, probably via regulating the transcription of several auxin signaling components and MYB transcription factors previously identified as required for fiber initiation. These results indicate that VINs may indirectly mediate root elongation by regulating the concentration of Hex to mediate the transcription of MYBs and auxin metabolism, including biosynthesis, transport and perception. In this regard, we found that many *MYB* genes annotated to function in cell division and many auxin-metabolism-related genes annotated to function in root development were differentially expressed in the leaves of *CsINV5*-OE plants under normal conditions (Additional file 5: Table S6). Whether and how these genes interacted with Hex to control cell elongation and lateral root growth remains to be tested in the future.

### *CsINV5* enhanced cold tolerance in *CsINV5*-OE plants mainly through the osmotic-dependent pathway

Based on the above, the mechanism of *CsINV5* response to low temperature was evaluated by overexpression analysis. In the present study, we found the VIN activity were decreased both in WT and *CsINV5*-OE plants when they exposed to low temperature condition, but *CsINV5*-OE plants showed higher VIN activity than the WT plants both under normal temperature and cold conditions (Fig. 6). As a result, the higher VIN activity promotes the Fv/Fm values and TSS contents, especially Glc and Fru increased in *CsINV5*-OE plants than in WT plants under cold conditions (Fig. 6).

However, the functions of *VIN* genes are varied in different plant species. As Chen et al. [71] reported that the VIN activities in two evergreen woody plants were decreased as the temperature declined in winter. However, in our study, we found the expression of *CsINV5* in tea plant was induced during CA with decreasing temperature (Fig. 2b and c), and a similar results was also obtained by short-term cold treatment (Additional file 1: Figure S4b), but the transcriptions of two VIN genes (*AtVIN1* and *AtVIN2*) corresponding to native VIN activity was reduced in *Arabidopsis* under low temperatures condition (Additional file 1: Figure S4a). Similarly, *AtVIN2* gene inhibited can significantly promote the accumulation of proline content, and then increase the cold tolerance of the transgenic *Arabidopsis* under cold condition [72]. In contrast, we found that overexpression of *CsINV5* in transgenic *Arabidopsis* could improve the cold tolerance of *CsINV5*-OE plants, and promote the expressions of two rate-limiting enzyme genes in Pro biosynthesis, *P5CS1* and *P5CS2*, suggesting that Pro content is higher in *CsINV5*-OE plants under cold condition. In a word, we suggest that the mechanisms of *VIN* genes in responding to low temperature are varied in different species, some genes with high expression may enhance the cold resistance of the plant, but the others may promote the cold resistance with low expressions.

Soluble sugars, especially Suc, Glc, and Fru, not only act as energy and carbon sources for plant growth and development but also function as signaling molecules, osmoprotectants and antioxidants to support abiotic stress responses [2, 73–77]. Therefore, the higher concentration of soluble sugars in *CsINV5*-OE plants under cold condition, may act as antioxidants to maintain ROS homeostasis, prevent membrane lipid peroxidation, and alleviate injury from low temperature. Accordingly, we found that transcription of some antioxidant biosynthesis-related genes was lower in *CsINV5*-OE plants than in WT plants (Additional file 1: Figure S5), indicating that *CsINV5*-OE plants may be less susceptible to freezing injury than WT plants due to higher sugar contents.

Suc and Glc act as signaling molecules that mediate auxin biosynthesis to regulate plant cell division and expansion [78], and this interaction also participates in responses to environmental stimuli [79, 80]. *CsINV5*-OE plants showed increased expression of many DEGs related to auxin biosynthesis, metabolism, signaling and transport (Additional file 5: Table S6), and most of these genes, such as the members of the SAUR and GH3 families down-regulated by Glc treatment in *Arabidopsis* [81], were also down-regulated in *CsINV5*-OE plants under cold conditions, but whether and how Glc signaling interacts with auxin signaling in *CsINV5*-OE plants to support cold stress response requires further study. Glc signaling requires a sensor hexokinase (HXK), which sequentially regulates plant gene expression at the transcriptional, translational, and post-translational levels [2, 82, 83]. Among the DEGs, we found a hexokinase gene, *AtHXK2*, up-regulated in *CsINV5*-OE plants under cold conditions (Additional file 5: Table S6); *AtHXK2* may mediate Glc signaling to improve cold tolerance in *CsINV5*-OE plants.

Osmotic stress and the associated oxidative stress are common consequences induced by drought, salinity and low temperature [32, 84]. In plants, Suc hydrolyzed into Glc and Fru by VINs is thought to double its osmotic contribution, sustaining favorable cellular turgor and facilitating water influx [20, 45, 58, 69]. Higher contents of Glc and Fru, associated with higher ratios of Hex/Suc, were found in *CsINV5*-OE plants under both normal and cold conditions (Fig. 6) and may help sustain favorable osmotic potential, maintain normal water influx, protect plasma membrane integrity against water deprivation or oxidation, and alleviate injury caused by low temperature. Stronger evidence supporting this hypothesis was found in the RNA-seq results, where the expression of two rate-limiting enzymes in Pro biosynthesis, *P5CS1* and *P5CS2*, were up-regulated in *CsINV5*-OE plants under cold conditions, suggesting that Pro content is higher in *CsINV5*-OE plants. Under osmotic stress, the osmoprotectant Pro is rapidly synthesized in cells to reduce water potential and prevent water deprivation [85, 86]. Moreover, GO enrichment analysis results showed that the transcription of some DEGs reported to respond to osmotic stress, such as *AtLEA3*, *COR413-PM1* and *COR15B*, were up-regulated in *CsINV5*-OE under cold conditions, suggesting that these genes as well as *P5CS1* and *P5CS2* contributed to maintaining osmotic balance to prevent *CsINV5*-OE plants from low-temperature injury. Thus, the stable osmotic potential in *CsINV5*-OE plants may attenuate the perception of calcium ion signals, and then a series of cold-related genes located downstream of the calcium signaling pathway, including CDPK, MAPK, CBF/COR, and ABA signal-transduction-related genes, would maintain approximately normal transcription levels (Table 2, Additional file 5: Table S6). To summarize, the improved cold resistance of *CsINV5*-OE plants derives from many metabolic changes, but the osmotic-dependent pathway may be an important factor.

## Conclusions

In conclusion, we found the LTRE-related and WBOXHVISO1 motifs within the promoter of *CsINV5* were the core *cis*-elements in response to low temperature and sugar treatment; the former participated mainly in response to cold stress, and the latter positively mediated the expression of *CsINV5* under both sugar and cold conditions. In addition, overexpression of *CsINV5* in *Arabidopsis* promoted taproot and lateral root elongation through an osmotic-independent pathway and enhanced cold tolerance mainly through the osmotic-dependent pathway.

## Methods

### Plant materials and treatments

Four-tea cultivars (DMB, LJ43, ZN12, and ZN113) that have been grown for 15 years from the same farm (TRI, CAAS, N30°10′, E120°5′) as reported by Wang et al. [40] were used for present study. These selected cultivars were used for comparative expression analysis of *CsINV5* during natural cold conditions. For natural CA analysis, the third to the fifth mature and healthy leaves from more than 10 randomly selected tea plants of each variety were collected and mixed together between 9:30 a.m. and 10:30 a.m. on each collection day. Three independent biological replicates were used for each sample.

The *Arabidopsis* ecotype Columbia-0 was used as wild-type (WT) and to generate the transgenic lines containing the promoter or the ORF of *CsINV5*. The seedlings of the selected transgenic lines and wild plants were firstly grown on 1/2MS medium (1.5% Suc, 0.8% Agar, pH 5.7) for 7 d, then transplanted to a soil mixture (3: 2: 1 peat moss: vermiculite: perlite) for 20 d in the growth chamber with photoperiod (10 h of light at 22 °C and 14 h of dark at 20 °C) and 100 μmol/m^2^ s as described by Wang et al. [40]. Seeds of WT and T_3_ homozygous transgenic lines were sterilized and vernalized, and the control vector homozygous transgenic lines were omitted as they have the same morphological as the WT as described by Wang et al. [40].

### Sugar and cold treatment in tea plants

For sugar treatment, 1-year-old plants raised from cuttings of the major local cultivar ‘LJ43’ were used hydroponically. Before proceeding, the hydroponic cuttings were cultured in the greenhouse with photoperiod (14 h of light and 10 h of dark at 23 °C, 75% of humidity) and a light intensity of 12, 000 Lux as described by Qian et al. [38]; then, 3% Suc, 3% Glc, 3% Fru and 3% Man were added into the nutrient solution. All sugar treatments were carried out for 2 days at 25 °C, and the second and/or third mature leaves from the terminal bud were sampled and mixed together at 3, 12, 24, and 48 h post-treatment. Thereafter, all sugar treatments were continued for 4 days at 4 °C, and the mature leaves were collected at 3, 12, 24, 48, 72 and 96 h, respectively. Finally, all the hydroponic cuttings with sugar treatments were returned to 25 °C for 1 day, and the mature leaves sampled at 6 h and 24 h. All samples were quickly frozen in liquid nitrogen and stored at − 80 °C until used. Three independent biological replicates were performed at each sampling time point, and each replicate contained four cuttings. The nutrient solution formulation is listed in Additional file 6: Table S7.

### Phylogenetic and conserved domain analysis of CsINV5

MEGA 6.0 software and the Neighbor-Joining method with 1000 bootstrap replicates were used to explore the phylogenetic relationships of CsINV5 with known VINs and CWINs, and DNAMAN software was used to investigate the characteristic amino acid sequences of CsINV5 and the known AIs. The amino acid sequences of the AI proteins used for analysis are listed in Additional file 7: Table S8.

### Cloning and sequence analysis of the promoter of *CsINV5*

The genomic DNA used as the PCR template was extracted from the tea plant cultivar ‘LJ43’ with the cetyl trimethylammonium bromide (CTAB) method [87]. The 5′-flanking sequence of *CsINV5* was amplified using the Genome Walking Kit (TaKaRa, Dalian, China) following the manufacturer’s instructions. The gene-specific primer1 (GSP1) and gene-specific primer 2 (GSP2) of *CsINV5* were used for the first and the second round of PCR, respectively. Subsequently, the amplified and purified PCR product was cloned into the *pMD18-T* vector (TaKaRa, Dalian, China) and sequenced. Finally, the PlantCARE website was used to predict *cis*-acting regulatory elements. All specific primers mentioned above are listed in Additional file 6: Table S9.

### Construction of vectors and plant transformation, and sugar and cold treatments in transgenic plants

To fuse the promoter of *CsINV5* into the GUS-plus vector (*pBI101::GUS*), 5′-deletions of the *CsINV5* promoter at − 1154, − 508, and − 342 were amplified by PCR using different forward primers (V5PF1, V5PF2 and V5PF3) and a single reverse primer, V5PR (Additional file 6: Table S9). The amplified and purified PCR product was cloned into the *pMD18-T* vector and sequenced, and then the promoter fragments were sub-cloned into the GUS-plus vector to form *P1154CsINV5::GUS*, *P508CsINV5::GUS* and *P342CsINV5::GUS* according to the restriction endonuclease method.

To construct the overexpression vector, the Gateway technology was used [88]. In brief, the ORF of *CsINV5* without a stop codon was amplified with the primer pair V5F and V5R (Additional file 6: Table S9), and then the fragment was fused into the entry vector *pENTR/D-TOPO* (Invitrogen, CA, USA) according to the manufacturer’s instructions and sequenced (‘*pENTR-CsINV5*’). Subsequently, the *CsINV5* ORF was transferred from the entry construct into the destination vector, *pH7FWG2*, using the LR Clonase II enzyme mix (Invitrogen, Carlsbad, CA, USA) [89].

These recombined vectors as well as the empty vector (*pBI101::GUS*) were introduced separately into *Agrobacterium tumefaciens* strain *GV3101* and transformed into *Arabidopsis* via *Agrobacterium*-mediated transformation [90].

To explore the cold tolerance of the *CsINV5*-OE plants, the sterilized and vernalized seeds of both WT plants and *CsINV5*-OE plants (OE5–9, OE5–12 and OE5–15) with different transcript abundances of *CsINV5* were grown on 1/2 MS medium for 1 week, and then transplanted to soil mixture consisting of 3: 2: 1 peat moss: vermiculite: perlite for 3 weeks. For the cold treatment, both WT plants and *CsINV5*-OE plants were treated at 4 °C for 6 d followed by a mild cold stress (− 1 °C) for 2 d without change the light time and intensity. Leaves were collected after 6 d of 4 °C treatment and 2 d of − 1 °C treatment to measure INV activity, and sugar contents, including total soluble sugar (TSS), Suc, Glc and Fru. Four independent biological replicates were performed, and each replicate contained four seedlings. In order to measure electrolyte leakage (EL), both WT plants and *CsINV5*-OE plants were treated at 4 °C for 6 d, and then exposed to − 6 °C for 8 h, as described by Rohde et al. [91]. Three independent biological replicates were performed, and each replicate contained five seedlings.

To explore the function of the promoter of *CsINV5* under low temperature and sugar treatment conditions. We detected the GUS activity of the T_1_ positive promoter::GUS transgenic *Arabidopsis* by GUS staining firstly, and then three transgenic lines with relatively higher GUS activity were selected from each construct for the following experiments. The sterilized and vernalized seeds of WT plants and four independent transgenic lines of pBI101::GUS (empty vector), P1154CsINV5::GUS, P508CsINV5::GUS and P342CsINV5::GUS were sown onto a square plate with 1/2 MS medium without Suc and grown vertically for 4 d, and then the seedlings were transplanted onto a new square plate with either 1/2 MS medium without Suc or 1/2 MS medium supplemented with 3% Suc for another 7 d of growth. The seedlings in each treatment were kept at normal temperature or 4 °C for 3 d and then collected for the histochemical assay. Four independent biological replicates were performed, and each replicate contained eight seedlings.

For tissue-specific analysis, the tissues of the three independent T_3_ homozygous transgenic lines P1154CsINV5::GUS during different developmental periods were used to investigate GUS activity, including the leaves, roots, stems, flowers and siliques.

All fresh samples of transgenic *Arabidopsis* containing the promoter of *CsINV5* as mentioned above were immersed in X-Gluc solution (Fermentas, Canada) for 12 h dark incubation at 37 °C, then rinsed in 70% ethanol several times. The preparations were studied with a stereomicroscope (Olympus Corporation, Japan), and the photographs were captured with an industrial digital camera (Oplenic Corporation, China).

### Root development assay, and Fv/Fm, EL, sugar content and INV activity measurements

The sterilized and vernalized seeds of WT plants and *CsINV5*-OE plants were vertically germinated on 1/2 MS medium with 3% Suc and 1% agar for 4 d. Two seedlings of each genotype with similar growth states, representing one biological replicate, were transferred onto a new square plate and grown vertically in the same growth chamber. The root lengths were measured with a ruler at the same time every day and record the data, a total of 7 d were proceed. Finally, the phenotypes were captured with a scanner (Epson Perfection V700 Photo, Indonesia) after 7 d of development. Twelve independent biological replicates of each type of *Arabidopsis* were performed.

The EL was measured as described by Wang et al. [40]. To explore the maximum quantum efficiency of photosystem II (Fv/Fm), both WT plants and *CsINV5*-OE plants were allowed recovery growth under normal conditions for 1 day after spending 2 d at − 1 °C, and the Fv/Fm was measured under different temperature conditions. Before measurement, all plants were dark adapted for 40 min, and then the Fv/Fm was detected by a photon system instrument (Open FluorCam FC 800-O, Drasov, Czech Republic). Four independent biological replicates of each genotype were performed, and each replicate contained three seedlings.

For the sugar content assay, the collected rosette leaves from WT plants and *CsINV5*-OE plants were ground with liquid nitrogen. Then, 0.1 g powder was extracted with 1.0 mL distilled water and ultrasonic treatment for 30 min and subsequently shaken at 800×g for 30 min. After centrifuging at 12, 000×g for 10 min, the supernatants were used for TSS, Suc, Glc and Fru measurements with a corresponding sugar measurement kit (Suzhou Comin Biotechnology, Suzhou, China), respectively.

INV activity was assayed as described by Tomlinson et al. [92] and Wang et al. [45]. A total of 0.2 g collected leaves of each sample both WT and *CsINV5*-OE plants from different temperatures were used.

### RNA-seq analysis

Four-week-old WT and *CsINV5*-OE plants (OE5–15) were grown at 4 °C for 6 d, and then the temperature was reduced to − 1 °C for 2 d before sampling the leaves. Leaves at the 22 °C were sampled as controls. All samples were snap frozen in liquid nitrogen and stored at − 80 °C until RNA isolation. Three independent biological replicates were performed at each temperature, and each replicate contained four seedlings.

For RNA-seq analysis, the total RNA of each sample was extracted as described by Wang et al. [40]. RNA concentration and integrity were measured using the NanoDrop 2000 (Thermo Scientific, USA) and RNA Nano 6000 Assay Kit on the Agilent Bioanalyzer 2100 system (Agilent Technologies, CA, USA). Thereafter, 1 μg RNA was used to construct each sequencing library with the NEBNext Ultra RNA Library Prep Kit for Illumina (NEB, USA) following the manufacturer’s recommendations, and index codes were added to attribute sequences to each sample. Subsequently, the index-coded samples were clustered on a cBot Cluster Generation System, and the library preparations were sequenced on an Illumina HiSeq Xten platform. After sequencing, clean data were obtained by removing reads containing adapter, reads containing poly-N and low-quality reads from the raw data. Gene function was annotated based on the following databases: Nr (NCBI non-redundant protein sequences), Nt (NCBI non-redundant nucleotide sequences), Pfam (Protein family), KOG/COG (Clusters of Orthologous Groups of proteins), Swiss-Prot (manually annotated and reviewed protein sequence database), KO (KEGG Ortholog database), and GO (Gene Ontology). Gene expression levels were estimated by fragments per kilobase of transcript per million fragments mapped (FPKM). Differential expression analysis of two conditions/groups was performed using the DESeq R package (1.10.1), and the genes with an adjusted False Discovery Rate (*FDR*) < 0.05 according to DESeq were considered differentially expressed. GO enrichment analysis of the differentially expressed genes (DEGs) was performed by a web database (http://omicslab.genetics.ac.cn/GOEAST/index.php). For co-expression cluster analysis, Perl, R language and the cluster package were used to construct the K-means tree.

### Quantitative real-time RT-PCR (qRT-PCR) analysis

For qRT-PCR analysis in the tea plant and *Arabidopsis*, total RNA was extracted as described by Qian et al. [38] and Wang et al. [40], respectively. First-strand cDNA was reverse transcribed according to the user manual in the PrimeScript™ RT reagent kit with gDNA Eraser (TaKaRa, Otsu, Japan), and the qRT-PCR program was performed on a Roche 384 real-time PCR machine (Roche). The qRT-PCR reagents were the following: a total of 10 μL of reaction mixture, which included 5 μl SYBR Premix Ex *Taq*, 0.8 μl sense/antisense primers, 1 μl cDNA and 3.2 μl distilled water. The qRT-PCR program was performed as follows: 95 °C, 15 s; 94 °C, 5 s and 58 °C, 30 s for 40 cycles; then a melting curve. *CsPTB* [93] and *AtEF* (At5G19510) [94] were used as reference genes for quantifying the expression levels of the target genes according to the method of 2^-ΔCt^ or 2^−ΔΔCt^ [95]. The quantitative analysis of each RNA sample was repeated at least three times, and the representative data are expressed as the mean values ± standard error (± SE). The primers used in qRT-PCR are listed in Additional file 6: Table S10. For RNA-seq analysis, we calculated the log2 value by using the FPKM data of each sample, then the log2 value of each sample under different temperature conditions was normalized to WT samples under normal-temperature conditions; the data are shown in a bar chart with the qRT-PCR results.

### Statistical analysis

Statistical differences in various physiological or molecular indicators between WT plants and *CsINV5*-OE plants under different conditions were tested by a one-way Analysis of Variance (ANOVA) based on Tukey’s HSD test and/or Fisher’s least significant difference (LSD) test at significance levels of *p* < 0.05, *p* < 0.01 and *p* < 0.001, and the WT plants and *CsINV5*-OE plants act as factor and the data of various physiological or molecular indicators act as dependent variables.

## Additional files

Additional file 1: **Figure S1.** Homologous analysis of ten SAIs protein sequences. **Figure S2.** Promoter deletion analysis of *CsINV5* under Glc, Fru and different temperature conditions. **Figure S3.** Histochemical GUS staining of WT plants and transgenic *Arabidopsis* with the empty vector (*pBI101::GUS*) under Suc and different temperature exposure. **Figure S4.** Expression analysis of *AtVIN1*, *AtVIN2* in *Arabidopsis*, and *CsINV5* in tea plant under low temperature condition. **Figure S5.** Expression detection of selected transcripts by RNA-Seq and qRT-PCR. **Figure S6.** Partial co-expression patterns of DEGs between OE and WT plants in normal and cold conditions (*FDR* < 0.05, K-means = 10). **Figure S7.** Prokaryotic expression analysis of CsINV5. (DOCX 10071 kb)

Additional file 2: **S1. ** The genome DNA sequence of *CsINV5*. 1154 bp promoter sequence and 7 exons have been highlighted: (i) stress signaling (ARE, fully outlined in red; HSE, double outlined in red; TC-rich repeats, dot outlined in red; LTR, viridity highlighted; MYC, viridescence highlighted); (ii) light signaling (MRE, navy highlighted; I BOX, violet highlighted; L BOX, pink highlighted; SP1, underlined); (iii) Hormones signaling (ERE, blue highlighted; TCA-element, green highlighted); (iv) sugar repression (SRE, highlighted in deep yellow) and activation (WBOXHVISO1, highlighted in gray). The CAAT-box and TATA-box are yellow highlighted. PCR primer sequences highlighted in bold and italic. The exons were showed by red font. (DOCX 32 kb)

Additional file 3: **Table S1.** RNA-seq data and quality control. (XLSX 11 kb)

Additional file 4: **Table S2-S5. Table S2. ** Sixty-six significant GO terms (FDR < 0.05) mapped by 266 up-regulated DEGs between *CsINV5*-OE and WT plants. **Table S3.** Fifty-five significant GO terms (FDR < 0.05) mapped by 402 down-regulated DEGs between *CsINV5*-OE and WT plants. **Table S4.** Sixty-two significant GO terms (FDR < 0.05) mapped by 1248 up-regulated DEGs between *CsINV5*-OE and WT plants in cold conditions condition. **Table S5.** Sixty-eight significant GO terms (FDR < 0.05) mapped by 1479 down-regulated DEGs between *CsINV5*-OE and WT plants in cold conditions condition. (XLSX 27 kb)

Additional file 5: **Table S6. ** Partial DEGs (FDR < 0.05, log2FC) in *CsINV5*-OE plants compared to WT plants in normal and cold conditions. (XLSX 21 kb)

Additional file 6: **Table S7.** Nutrient solution formulation. **Table S9.** Primer sequences used in promoter cloning and vector construction. **Table S10.** Primer information used in qRT-PCR detection. (DOCX 22 kb)

Additional file 7: **Table S8.** Amino acid sequences of the SAI proteins used for conserved domain analysis. (XLSX 13 kb)

## Abbreviations

CA: Cold acclimation
CDPK: Calmodulin-dependent protein kinases
CIN: Cytoplasm invertase
CWIN: Cell wall binding invertase
EL: Electrolyte leakage
GUS: β-glucuronidase
Hex: Hexose
INV: Invertase
MAPK: Mitogen-activated protein kinase
TSS: Total soluble sugar
VIN: Vacuolar invertase
