# Supplementary material for: CsINV5, a tea vacuolar invertase gene enhances cold tolerance in transgenic Arabidopsis
Source: BMC Plant Biol. 2018 Oct 11;18:228. doi: 10.1186/s12870-018-1456-5 (PMC6182829; doi:10.1186/s12870-018-1456-5)
Supplement: Supplementary file 1 — Figure S1. Homologous analysis of ten SAIs protein sequences. Figure S2. Promoter deletion analysis of CsINV5 under Glc, Fru and different temperature conditions. Figure S3. Histochemical GUS staining of WT plants and transgenic Arabidopsis with the empty vector (pBI101::GUS) under Suc and different temperature exposure. Figure S4. Expression analysis of AtVIN1, AtVIN2 in Arabidopsis, and CsINV5 in tea plant under low temperature condition. Figure S5. Expression detection of selected transcripts by RNA-Seq and qRT-PCR. Figure S6. Partial co-expression patterns of DEGs between OE and WT plants in normal and cold conditions (FDR < 0.05, K-means = 10). Figure S7. Prokaryotic expression analysis of CsINV5. (DOCX 10071 kb) [file 12870_2018_1456_MOESM1_ESM.docx]

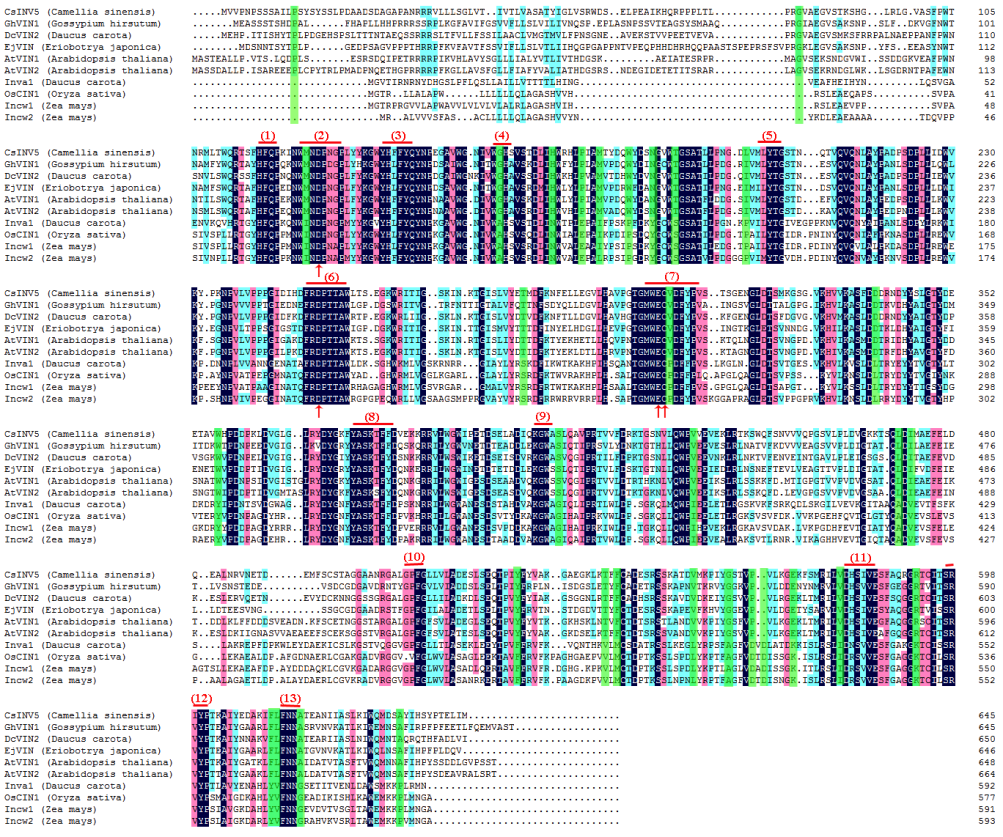


**Fig. S1** **Homologous analysis of ten SAIs protein sequences**. Thirteen conserved domains that reported by Ji et al. [47] are shown in red lines with the numbers. Four arrows showed the enzyme active site residues proposed by Alberto et al. [46]. All of the single amino acid residues contained in the green boxes probably can divide SAIs into VINs and CWINs.


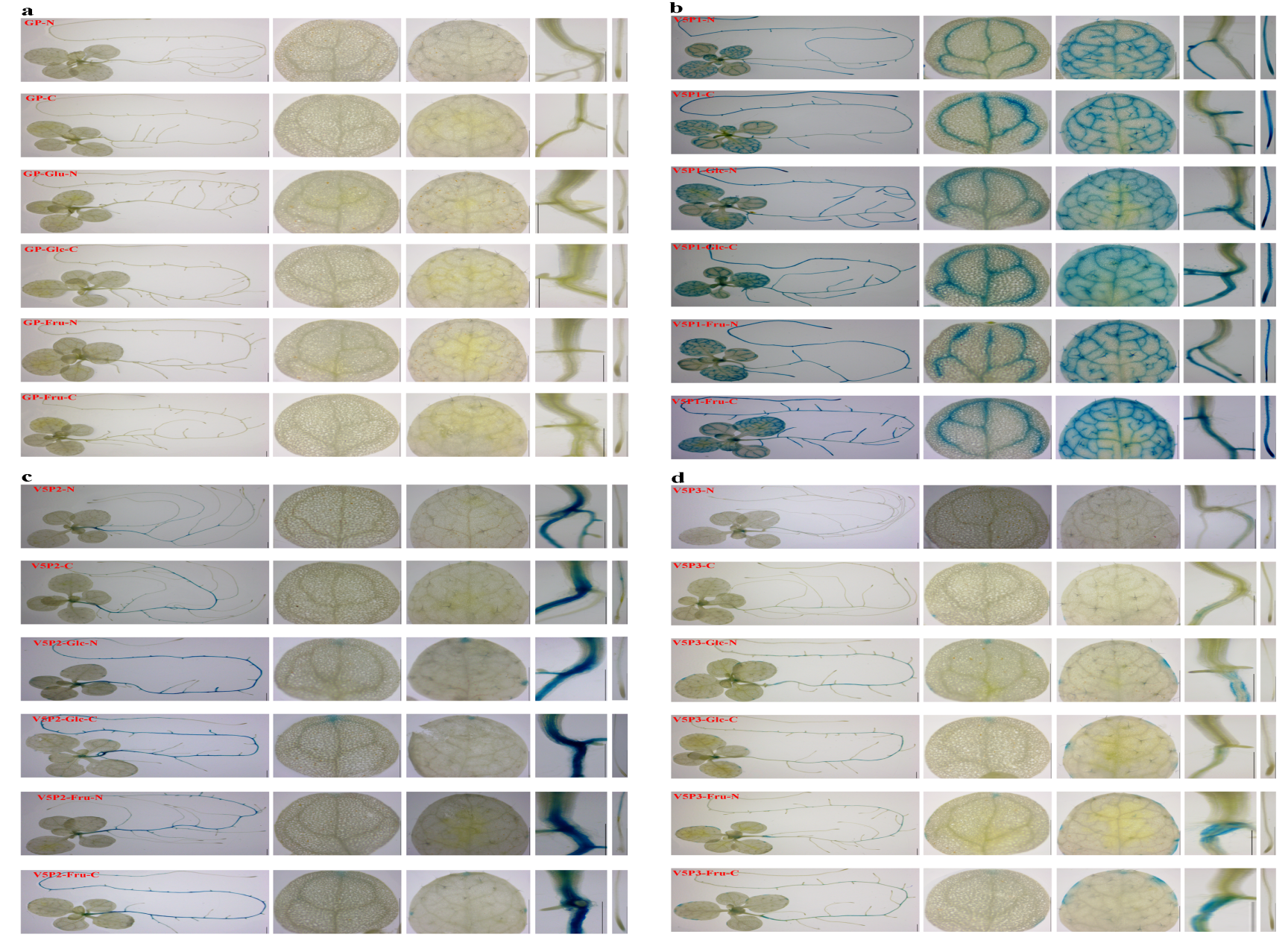


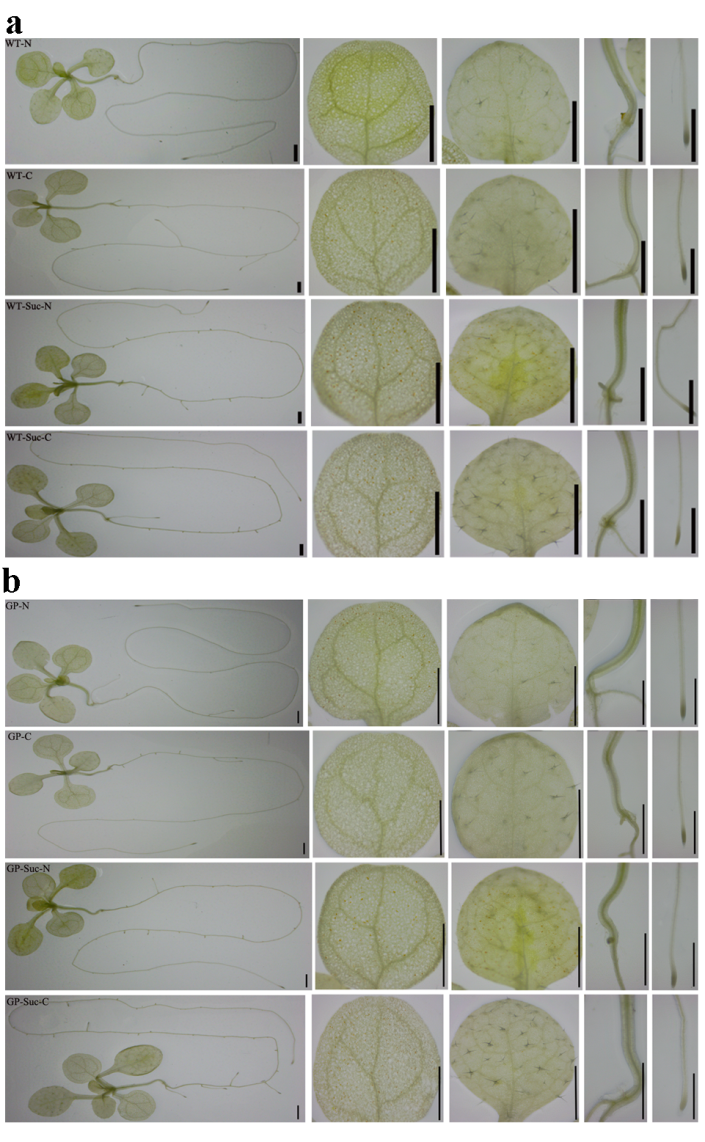
**Fig. S2 Promoter deletion analysis of *CsINV5* under Glc, Fru and different temperature conditions**. **a-d** Histochemical GUS staining of transgenic *Arabidopsis* with the empty vector (pBI101::GUS), P1154CsINV5::GUS, P508CsINV5::GUS, and P342CsINV5::GUS under normal-temperature and Sugar-deleted conditions (GP-N, V5P1-N, V5P2-N, and V5P3-N), low-temperature and Sugar-deleted conditions (GP-C, V5P1-C, V5P2-C, and V5P3-C), normal-temperature and Glc-added or Fru-added conditions (GP-Glc-N, V5P1-Glc-N, V5P2-Glc-N, V5P3-Glc-N, and GP-Fru-N, V5P1-Fru-N, V5P2- Fru-N, V5P3- Fru-N), and low-temperature and Glc-added or Fru-added conditions (GP-Glc-C, V5P1-Glc-C, V5P2-Glc-C, V5P3-Glc-C, and GP-Fru-C, V5P1-Fru-C, V5P2- Fru-C, V5P3- Fru-C), respectively. Scale bars = 1 mm.


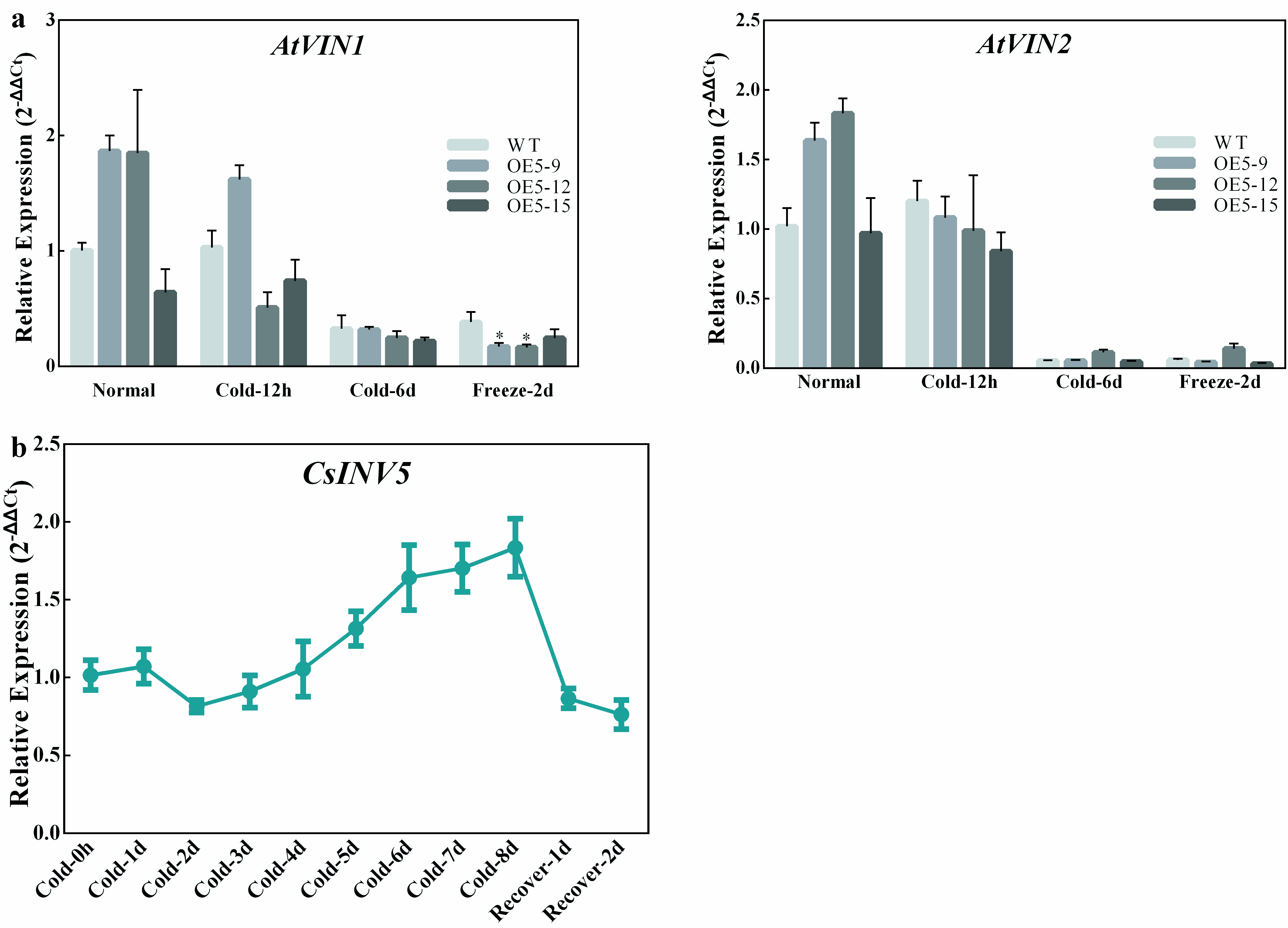
**Fig. S3 Histochemical GUS staining of WT plants and transgenic *Arabidopsis* with the empty vector (*pBI101::GUS*) under Suc and different temperature exposure**. **a** WT-N and GP-N represent normal-temperature and Sucrose-deleted conditions; WT-C and GP-C represent low-temperature and Sucrose-deleted conditions; **b** WT-Suc-N and GP-Suc-N represent normal-temperature and Sucrose-added conditions; WT-Suc-C and GP-Suc-C represent low-temperature and Sucrose-added conditions. Scale bars = 1 mm.

**Fig. S4 Expression analysis of AtVIN1, AtVIN2 in *Arabidopsis*, and *CsINV5* in tea plant under low temperature condition**. **a** Expression patterns of *AtVIN1*, *AtVIN2* genes in Arabidopsis under normal temperature (Normal), 4°C for 12 h and 6d (Cold-12h, Cold-6d) and -1°C for 2 d(Freeze-2d); **b** Expression patterns of *CsINV5* in tea plant under 4 °C for 8 d (Cold-0-8 d), and then recovered to normal temperature for 2d (Recover-1d, 2d).


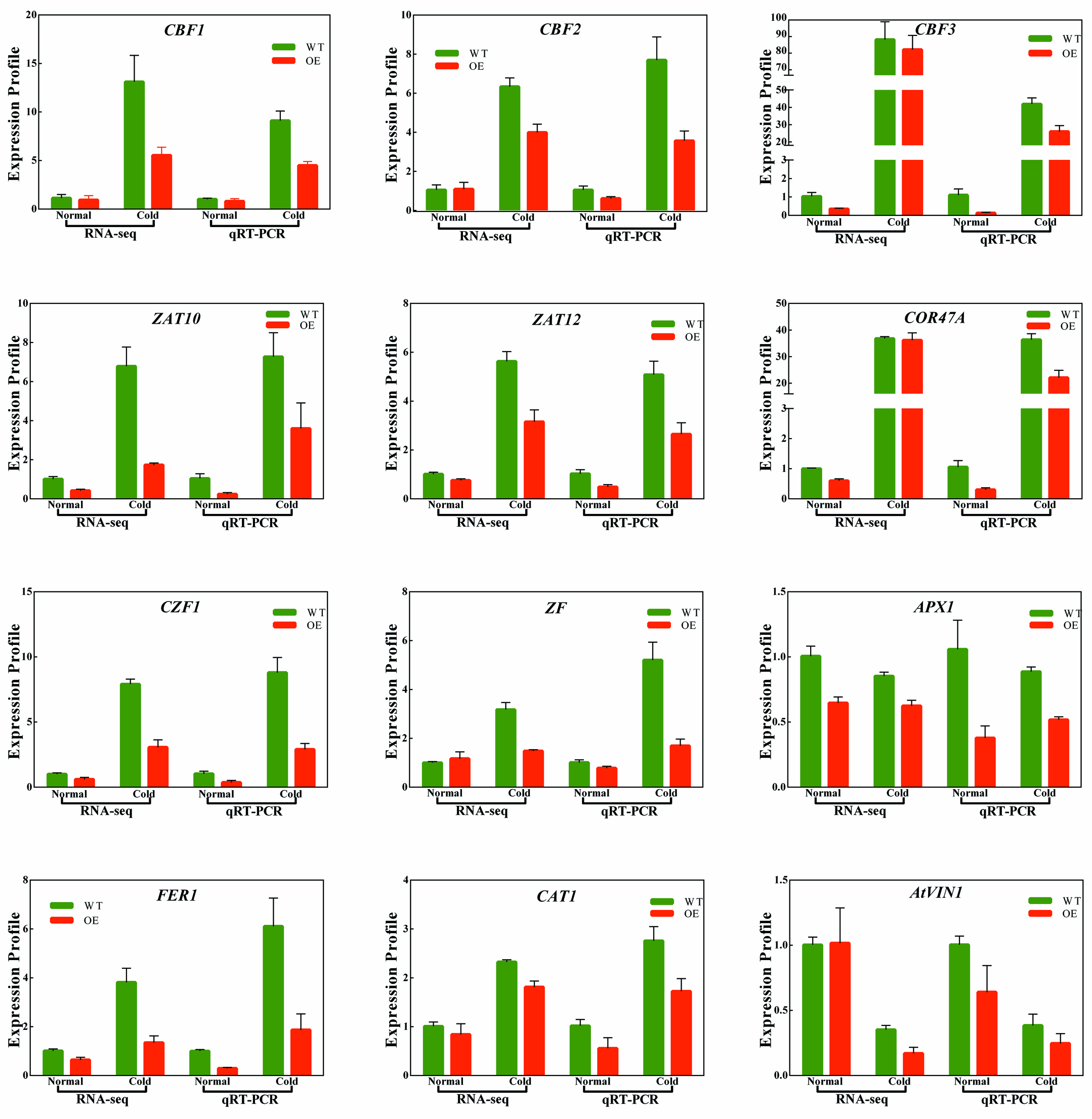


**Fig. S5 Expression detection of selected transcripts by RNA-Seq and qRT-PCR**.

**Fig. S6**
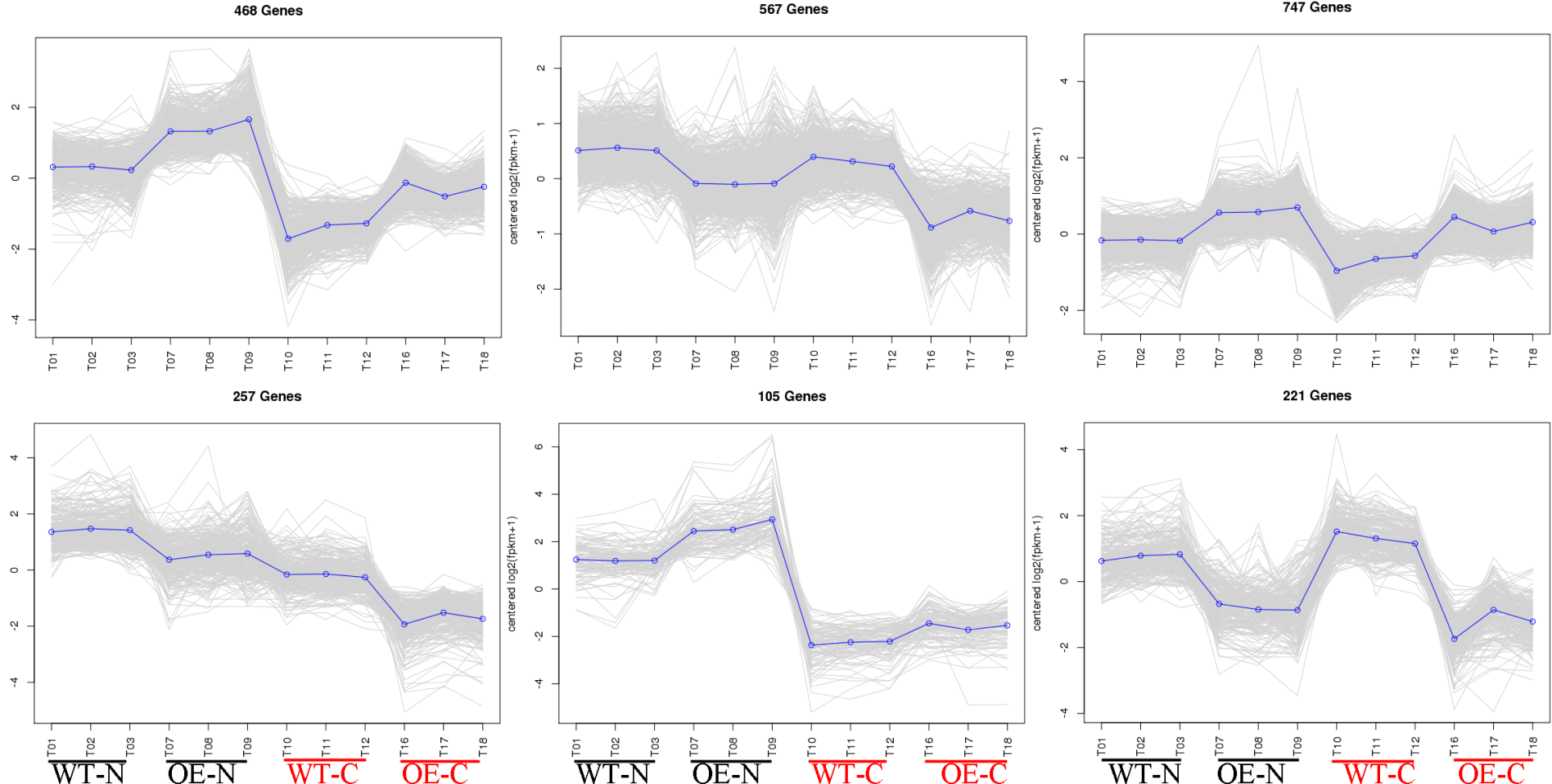
 **Partial co-expression patterns of DEGs between OE and WT plants in normal and cold conditions (*FDR* < 0.05, K-means = 10)**.


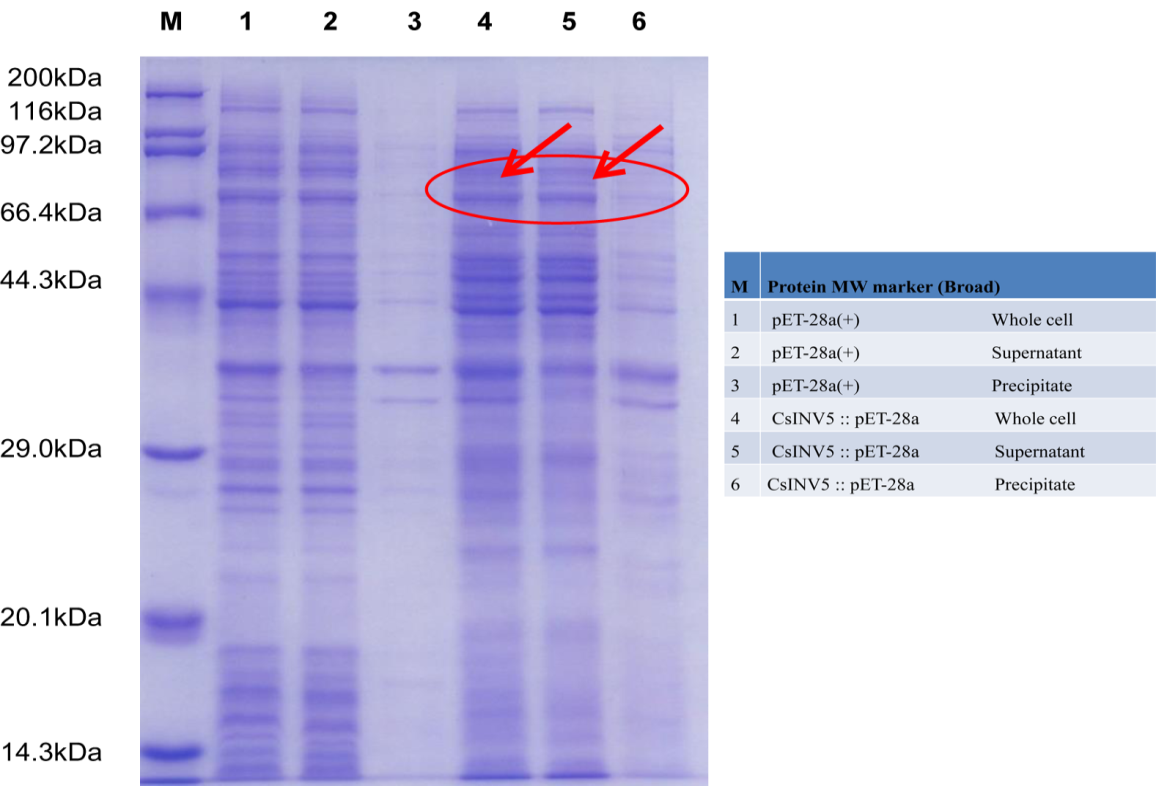


**Fig. S7 Prokaryotic expression analysis of CsINV5**. M: protein MW marker; 1: The Bacteria protein band of whole cells with the empty vector pET-28a; 2: The Bacteria protein band of supernatant with the empty vector pET-28a; 3: The Bacteria protein band of precipitate with the empty vector pET-28a; 4: The Bacteria protein band of whole cells with the recombinant plasmid of *CsINV5*; 5: The Bacteria protein band of supernatant with the recombinant plasmid of *CsINV5*; 6: The Bacteria protein band of precipitate with the recombinant plasmid of *CsINV5*.
