# Supplementary material for: CsINV5, a tea vacuolar invertase gene enhances cold tolerance in transgenic Arabidopsis
Source: BMC Plant Biol. 2018 Oct 11;18:228. doi: 10.1186/s12870-018-1456-5 (PMC6182829; doi:10.1186/s12870-018-1456-5)
Supplement: Supplementary file 2 — S1. The genome DNA sequence of CsINV5. 1154 bp promoter sequence and 7 exons have been highlighted: (i) stress signaling (ARE, fully outlined in red; HSE, double outlined in red; TC-rich repeats, dot outlined in red; LTR, viridity highlighted; MYC, viridescence highlighted); (ii) light signaling (MRE, navy highlighted; I BOX, violet highlighted; L BOX, pink highlighted; SP1, underlined); (iii) Hormones signaling (ERE, blue highlighted; TCA-element, green highlighted); (iv) sugar repression (SRE, highlighted in deep yellow) and activation (WBOXHVISO1, highlighted in gray). The CAAT-box and TATA-box are yellow highlighted. PCR primer sequences highlighted in bold and italic. The exons were showed by red font. (DOCX 32 kb) [file 12870_2018_1456_MOESM2_ESM.docx]

**S1:** The genome DNA sequence of *CsINV5*. 1154 kb promoter sequence and 7 exons have been highlighted: (i) stress signaling (ARE,fully outlined in red; HSE, double outlined in red;TC-rich repeats, dot outlined in red; LTR, viridity highlighted; MYC, viridescence highlighted); (ii) light signaling(MRE,navy highlighted; I BOX, violet highlighted; L BOX,pink highlighted; SP1, underlined;); (iii) Hormones signaling (ERE, blue highlighted; TCA-element, green highlighted); (iv) sugar repression (SRE, highlighted in deep yellow) and activation (WBOXHVISO1, highlighted in gray). The CAAT-box and TATA-box are yellow highlighted. PCR primer sequences highlighted in bold and italic. The exons were showed by red font.

(-1154)***ACGACGAGAGAGACGTAGAAGAGA***GAAATCAGAATTAAGGAATAGTAGCGTCCCTACAGTAGTCTTTGCCATTTCGGAGTTAGAAAAAAAAATGAACCTAATAATTTTAAAAGTAATTATTGGGCTTTGATATTTATGTTTATGGAATACACCATGAATATGTTTTAAAAGTCTAAATTATTTTACTATTATTCAACAACTAATAATTTTATAGATATACTTAATATTTATATATATAATTTAAGCTTTAATTGCTGAGATTGGTGGATGTTTTTGTTCAAACTTGATTTAAATTTAATTCAAACTTTTAAATTTTTGTTCAAGTTTACACAAAATTAAAAAATATATTATCCAAACTTTAATATTTTTTAAATAAAAAACTCGGGTTAAACCCGGGCAAAATGAACCCTTATCGGCCAGTCCCACATAGCTTCGATTGGGAAGAAGGATTGGGTTAAGTTTGGGACGATTTTAGTTACAAAAAAATAAAATAGAAATTTTAGTTCAATAAGATAAAAATATTACATCAAATCTATAATTATTACATTCTTTCTCTCACAAAAAATTAAAAAATTCAAAAAATCTTTTCCAAACGAAACTTTTTAACCTCAAGCTAAAATAGTTAAAAAATTAAAAAGTTAAAAAA***TCAACTCACCTCCCAAACGAAT***CCAATCACTTTGTTTGGTTTTAAAGTTACAAGTGAATAATTTTCCTTTATGGAATAAATAACAAAATACAAAAAATAAAAATTTCAAAAATTATTTGTTAAAAAACTCAAAACAAGTGCTTAATTTTCCTTTCAACACATGTTA***AAGAATTGTTCAGCCTCACATTT***AAATGGAAAGTTGTTCATTCAATTCACAACTCTCTCTCTCTCTCTCTCTCTCTCTCTCTCTCCAAAACTCTCTCTCCAAAACTCTCTCTTTCTCTTCCAACCATATTATAAGCACGAAAACCAGCCAACCCTATTAACAGCCTCCCCGTAGTCCCGTTGGTCCGTAGGTCCATTTCTGGCAAATCAGAATCTGACTCACCCCACCTCTCTCTCTCTCTCTCTCTCTCTCTCTCTCTCTCTTATATATATATATATATACACCCCCTAAATCTCGATCCTCCTACACAAACAATCCAAACACTCCAAAAAGTCTCAAACC(+1)AT

GGTGGTTCCAAACCCATCTTCATCCGCCATTTTACCCTCCTATTCCTACTCATCTCTGCCCGATGCGGCCGATTCCGACGCCGGAGCTCCGGCGAATCGCCGCCGTGTCCTCCTGCTATCTGGGTTGGTGACAATCGTGACCCTGGTGGCTTCTGCCACTTACATTGGTCTCGTTTCGAGGTGGGACTCGGAGCTGCCGGAGGCCATCAAGCACCAGCGTCCTCCGCCGCTTACGCTGCCGCGTGGGGTGGCGGAAGGCGTCTCCACCAAGTCCCATGGCCTCCGTCTGGGGGTCGCATCCTTTCCTTGGACCAATCGCATGCTGACGTGGCAGAGAACATCCTTCCATTTTCAACCTAAAATTAATTGGATGAACG**(Exon1)**GTATTTACTTTTAATTTCTTTTTAATCAATCTGGTATATGCGCTTATCCACCCAAAAAAATCTAGTAGATACCACATAGGAGAGATTTATTATTTTGTATTTTATAATAAAAATAGTCATATAGACAATTTATATGTAATATTAGAGAAATTTATGCACAATAAAATAATAATTAGTAAAAAAAAATGGTATTAAATAAATTTCATATAAAATTTTTGGGCATAGAGTTAGGAAATGAAAAAAATTAGACTACAACCACACATAGAAAAATTTTAGATTACCAAAATAAATTAAAAGACACAAAATAATTTCAGTCATTTTTTAAAAATCTAAATTATACAAAAATATACCTGCCTAACCCCAATAAAATAATAAAACCGTTACATACCAAGACCTAAAATTAATTGGATGAACGGTATTTACTTGCTTTTAATTTCTTTTTAATCAATCTAGTAGACGCCTATCCACCCAAAAAATCTAGTAGATGCCACGTAGGAGAGATTTATTTATTTTTTTGTTTTTATGAACGGGAGAGATTTATTATTTGAAAAATTTACCCATAAGTCCTAGTTTTTGAAAAGTTGAACCATTTAGTCCTAAAAGTTTTCTTATGCTCCATTTAGTCCCATTTGCACATCAATCTAATGTTTGAAACATTAATCTGATGTTTGAAATCTAATGTTCGAAACATTAATCTGATGTTTGAAATCTAATGTTTGAAACATTAATCTGATGTTTGAAACATTGTAATGGGACCTTTCAGTCTATAAGAAAACTTTTAGGACTAAACAGTGTATAAGTTATTAATTTGAGATTTATTGGTGCATCCCCCTTATTATTTTGTATTTATAATTTAACAAAAGAATAATTTGATAAAAAGTTTATTTATTTTACATTGGAGTCTATAGAGGAGGAGGATTTTCTCCGGTTTAAAATTTAAGGAGATTGTTTGGATTAGTAGAATTTATTATGGGTCTCGTTTTAATAATTTAAATTGTTCATTTTGTCGGAATTTCGTGTATTTCATTAATAAAATAAATTAAATCGATCCAATAATTATAATTTGTTCAATTTATTATTTTTGTCATAAAAAAATATATGATTTATTTTTTAAAAACACAATCGTCATTTATTTTTTTAATAAAAAATATGAAAAATTGAACAAGTTATAACTATCGGGTCGATCTAATTTATTTACAAATAAAATATACAAAATAATTAATAAAATAAATGATTCAAATCGTTAAAATAGGACCCGTGTGACAGCCAATCAGGCCCTTCAATCCTCCTCCATGTAAATAAAGACACGTTACTTAGGAAGATATCTTGTTTGACACTTTCTATTTATGTGATTCTATTCAATCATTAAATGAGACTCGGTAAAAATGAGATTATCCCTTATAACACGTTTCTTTTATCTGTTTCATTCAATAAAAGGCGTTTATGGAATAAAGAATTAGGATTGACCAAAAAAAAAGCCTAGAGACCATTCCCAGTTTTGCTATGCTTGTTGTGATTTAATAAATTATAATAAAATCAATGATACGTAGCAATTTTTTATTTATAGTTTTTGAAGTTTGAGCACACATTGATGCAAGAATTGTTAAGATCAACAATGATAAACTCTTCTAAATTAATGAATTATAATGAACTCTAATGCCATGAAAAGTAAAAAAAAAAAAAATAAAAATACAGGACACATACATTACTATTACAGTGACACTCACACTCTACTAGAGCTATTAGAACTCTTTTATTAGAGTTTTAAAATATTTTTATTGTTAAGATTTAGGGAGTAAGATTATTATGAATGGTTATTGGATTGACAGAATTTTTGAAAAGTGAGTTTTAGGCTAAGTTTGGGATGTTAAAAAAAAATCAAAAAGTAAAAAGTCACTATCCTATTTAAGGGGTTGTTCGGGGTGTCCTAAATAAGTGCACTTTTTATCTTTTTAGACTAAAAAGTTGAGGAAAAAAATATGAATGTTTGGGTTAACTTTTTGACTTTTTATGGAAAAATGTGTAATAAAAAATTATAAAAATTAGTTTGTGGAGGAAAATTAGGATGGAGAGTTGTGCAACCGTAATTGTTGACTTTTGCTGAATAGTGTTAACTTTTTAACTTTTTGACTTTTTTTAAGTATCCCAAACTTGGCCTAAGTTACATCATCTTATATAAGTCACATCACCTCTTTACCACATCACTTCAAATTCAACTCATATTTTATAAAAAATAAAAAAATACACTTATTTAGAATATTCCAAATAACCCTTTATTAAGAAATGTCGTGAAATCTTTTCAATCTATGTAGTTTTTGTGTTTGATTATTTATGGGGTTTTTTTTTTTTTTTTTTTTGTAAAATCTTAATGACAGAGTAATTATTCTTGGTATGAGTAATTAACTGAATGTTGAATGCTTATTTTCATTTACTAATATATTTTAGTTTGACTTTTATTTATTTATTTATTTTTTTTCCGATTTCTTGTGCTGTCATTGCTATGGTGGATGAATCGTTTCTTCTGGTGCAGATCCCAACG**(Exon2)**GTATGCTTTTACATCTCTGCCCCTCTCTCCGCACCCACGTGTACACTTGTCTAATTAGATACAACGAGACTTTTTTTTTTTTTTTTTTTGCCCTCTCTTTAGGATTTAAATTACTATGCAACTGACAAATTTGCATCTAGAGTGACGTTTGGTAAGAAAAATGTTAAATATAGACTTGTTTAATTTGGGAATTGTCTGAGAAAATAGAGAGATTAATTTAGTAATTTAGTGAGATTAATTGTCTGAGAAAAATGTGACATTTAGTAATTTTTTAATAAAAATATATACAGAAAAATAGAAAGATTAATTTGATAATTTAAAATGATTTTTTGATTAGAAACTAAAGTTTCCAAAAAGTCTTTAGATATGTGTAGGTAGGGGGGAAAAAAGAAAAGAAATTTCAAAGAAAAAATTTACCTTTCATTTTTTATTTACAAATTTTTTTCTTTACCTTTAATTTTACTAATTTTTATGCAAATTCAACATTCAAACATTTCAAAAACATATGATCACGGTTGAAACCATCATTGTCATAAATAGTGGTGAAAACAATCAATTAAAAAGTTACAGTAAAATCACATTTTAATTTCTTATATCCACAATTCTTTCCCTTTCAACTTTAGAAGCATAAAAAGTTATCAATGGCGAGAATTTTGGATAGTTATTATTTTTTAATTTGGATTAGTGCAATACATTGGTTGGTCCACGCAATCACTTTCCAAGGAGAGTCTTTTGGACTTTAATAATCATAATTATTATTCATTTAAAAAGAGCACTAGTTGTACCAAAATTGTGTTGGATTTGTGGATTGACAAGAGCCACGTGGATGTGTCTAACAATTATGTCGATAGACAAAGTCAGATTGAAAGCGATTTGAACTGGACAAAAAGACAGGACATGTATTTACCTACTTATTTAAGAGTAATAATGCTAGGAAATTTAATTTTTTACATTAAATGTCCTTATCCATGACATGGCATTTACAAATGGCTCAAAGCAGGTGAATTTTAAGAATGACGTGGCATTTTTACCATTAATGATCGTTACACCTGTGCTTATTAAATGACATTAATTAGTGAACAAACAACCATAATTTCTTTTGCAATTTTTTTAAAAACGATTATTAATTTCAAATCTCAACATAAAATCCAAACATAATACATGATGTTATTTAAACTCATTTTTTTACAAATTAATATATTAAATAATCAAATTATAGAAATTTTTTTTTGATGAAAATAAGGGATAAATACACATGTGTAGGAGTTGAGTTAAGTCAATTTATATAGTTGGATTAACAATTTATGAACTGTATGCGAATCATCATATTATTTTTTGTCATCTAGTTTGTATAACTTACTTGTTCATTTTCTAAATTTGTACGAGTTGATCTGTACTTATAAATAAACATTCCCCCCTCTCCCTCCCTCCCTACCTAAAATACAGTTTACATGTCATCAATGCATTAAAGGATAATCAGGATTGTTCGGGATATAGATTGAGCTAATTTAGTTTATAAAAAGATAAAATAGATTAAAATATCACATATAAATCATAATCATTTAATTTTTTTTTCTTTCACAATCATCACATATAAATATAAAATAAAAAATAAAAAAATTATCTCAAATAAAACTTTTTTAATTAGACTAAAATAATTAAAAACTTATGCTAAAAAAATTTATCCCAAAGAAAGCCTCAAAAAAGACGATAAAAGAAGAAAACTCAAAGCGTAATTCATAACTTGAAGTAGAGGAAAACTTTAAATCAATTTTATTGTGAATGTTGTATTTGTTTTTTATAGGCTTAGTTTTATTTCTTTTGAGAGAGTAATTTATTTTTTGTTTATTTTTATTCTCCCTATAAAAGATATGTATTAAACTTTATTGTATATATATATTTTTAGTTAAAATATAACTTATAAAATTAAAAATAAACAAAAAAAAAGCCTTAATTAATGAGAACTTTATGATCCATGTTAAACATGATAGGTCCACTATACTACAAAGGGTGGTACCACTTCTTCTACCAATACAACCCAGAAGGTGCAGTATGGGGTAACATCGTGTGGGGTCATTCGGTATCAACAGACCTGATCCACTGGCGCCACCTCCCCATCGCGATGACCTACGATCAATGGTACGATAGCAACGGTGTCTGGACTGGGTCCGCTACCCTCCTCCCCAACGGTGATCTCGTCATGCTCTACACCGGATCAACCAATCAGACCGTGCAGGTCCAAAATCTAGCATACCCCGCCGACCCATCTGATCCCCTCCTCATCGATTGGGTCAAATACCCCAAAAACCCGGTTCTAGTACCCCCACCCGGAATTGATATCCACGACTTCCGTGACCCGACAACCGCCTGGCTCACATCTGAGGGCAAGTGGCGGATCACAATCGGGTCAAAAATCAACAAAACGGGCATTTCATTGGTTTATGAGACCATGGACTTTAAAAACTTCGAGCTCTTGGAAGGGGTGCTCCATGCGGTTCCGGGTACAGGTATGTGGGAGTGCGTGGACTTCTACCCGGTATCCACGAGTGGGGAAAACGGGTTGGACACGTCAATGAAGGGTTCTGGAGTGAAACATGTGGTGAAAGCTAGCTTTGATGATGATAGGAATGATTACTATTCTCTGGGGACGTATGATGAGGAAACAGCTGTTTGGCATCCGGATGATCCGAAATTGGATGTGGGTCTCGGGTTGAGATATGATTATGGAAAATTCTATGCATCGAAGACATTTTTTGATGTGGAGAAGAAGAGAAGGGTGTTGTGGGGTTGGATTCCGGAGACAGATAGCGAATTGGCCGACATTCAGAAGGGTTGGGCTTCTCTTCAGG**(Exon3)**TACATTCATATATTTCATACTCAATTTTTACATAAAGACAGTGATATTTATATAATTAAATATTATTTTTATTGGCACTTCGTGTAAAATGATAAATTTCAATGCAACACCCCCATTAAATTTGAATATATATCACACAATTCACTTTCTAGAAAAATATTTTTGTATTCCAAAAATTTCTGTGACGGTATGTAGTTTTTTCTTTCCTTACTACCCTCCTGCCCATAAATATGTCATACTTGTTGACAAAGATGCTAATGATTCAAACAGGCAGTTCCAAGGACAGTAGTGTTTGACAGGAAAACTGGAAGCAATGTACTTCAATGGCCAGTGGTGGAGGTTGAGAAATTACGAACGAAAAGCTGGCAATTTAGCAATGTGGTTGTCCAACCAGGCTCAGTCTTGCCCCTTGATGTCGGCAAAAAAACCTCTCA**(Exon4)**GGTTAGTATTACTTCTTCTACTTGCACAGAATCGATTCACAGAATGAGAGAGTTGTGTTTGAATTCTATGATTAGATTTTGTGAGCTCATTTTTCTTGTGGGAATGTAACAATTGGCTTATATTATCAATTGTGCATTGGCAATGAAACAGTTGGACATCATGGCCGAGTTTGAGCTCGATCAGGAGGCCTTGAACAGGGTGAATGAAACCGATGAGATGTTCAGTTGCAGCACTGCCGGTGGAGCAGCAAACCGGGGTGCTCTTGGACCGTTTGGTTTGTTGGTTCTTGCAGACGAGAGCCTTTCTGAGCAGACTCCCATTTACTTCTATGTTGCCAAAGGGGCTGAGGGCAAGTTGAAGACTTTCTTCTGTGCTGATGAATCAAGG**(Exon5)**TTTGTTACTCTTTTCATTGTGCACTTGAAACATACTCAATATTAATTAATATTTTTTGTCCTTGTTATAGTTTCATTGGTTATGAGTATTACCAAATTATATAAGCCCAACCTTATGAATTTTTTTTTTTTTTTTGAAAATTATATTACCTGCCCCTTGTTAACATTCATCTTGAAGTTAGAATCTCCATCGGAGAACCACCATTATTTTCATATAATTCGGATTTTTTCTCCAACGCGAAACATTGGCTTAGTATTATTAGAGGTTAGGACGGCAATGAGGCAGGTATGAGTCTTTGACCCCTGCCCTCCTCCACCCTTGTTTAGTGAATATTGGACCTTATCCGCCAGACTCTCCTCTCCCCCATCCTCGGCACTCCCCACATAATATAATCCAGTCTCTAACCCTACTTGCCCCGTTTAGTTTTATGACTTATATTTTTAGTATTGAATATTAATAATGTGGTTTTGATAGCTCTAGATGACAATGATTTAACATTGATTTAAGTTTGCGATCAGAGTCAAGGAAGAAAGAAAAGAGCTGCAATATCCAATAAAACAGAGAATAGAAGAGAAAAAAAAAAATCTTTAAAATCGTTTTATAATTATGTAATTTATATGTCAGGTTTTTTATAGTATGCATAATTGGGTCGGGTTACCCTCCCCAAACTCATTTTATTAGAATCGGGGAGGGTTGGGAACTTCTGGGTCAGGTTTTTCTTGCTATCCCTAATCAGAGGACTGTCTTTGGCATGAACAACAACTTGACTCGCCGATACAACTGTTTTTTGTGTAGGTCTTCAAAGGCAACAGATGTGATGAAACCAATTTATGGAAGCACAGTTCCAGTGCTCAAAGGAGAAAAGTTCAGCATGAGAATATTGGT**(Exon6)**ATGTTAAAAAAATCATTTTGCTTAGTAATAATAATTTGCTGCCTAAAATCCTAAAATTTATCGAAAATAAAATTTGGATTATTAAAATGCGGGTTCATTTTTAATTTAGGTGGATCATTCGATAGTAGAAAGCTTCGCGCAGCGCGGGAGGACATGCATAACTTCGCGGATATATCCAACGAAGGCCATCTATGAAGATGCAAAAATTTTCTTGTTCAACAATGCTACCGAGGCCAACATCATAGCCTCCCTCAAGATTTGGCAAATGGACTCTGCATATATTCATTCCTATCCCACTGAACTCATAATGTAA**(Exon7)**AAAAAAAAAAACACTTCTAATGCTCAATCATGTCTCTCCATTGCTCTCTACTTCAGATTGTGTACTGTCATTTTATCATCATTTTTTGCAGAAAAAAAGGAAAAAAAAAAA
