# Supplementary material for: CsINV5, a tea vacuolar invertase gene enhances cold tolerance in transgenic Arabidopsis
Source: BMC Plant Biol. 2018 Oct 11;18:228. doi: 10.1186/s12870-018-1456-5 (PMC6182829; doi:10.1186/s12870-018-1456-5)
Supplement: Supplementary file 6 — Table S7. Nutrient solution formulation. Table S9. Primer sequences used in promoter cloning and vector construction. Table S10. Primer information used in qRT-PCR detection. (DOCX 22 kb) [file 12870_2018_1456_MOESM6_ESM.docx]

**Table S7** Nutrient solution formulation.

| **Elements** | **Compounds** | **Concentration** |
| --- | --- | --- |
| **Macroelements**  **(mmolL^-1^)** | CaCl_2_ | 0.53 |
|  | NH_4_NO_3_ | 2 |
|  | KH_2_PO_4_ | 0.07 |
|  | K_2_SO_4_ | 0.6 |
|  | MgSO_4_·7H_2_O | 0.67 |
|  | A1_2_(SO_4_)_3_ ·l8H_2_O | 0.07 |
| **Microelements**  **(μmolL^-1^)** | EDTA·Na_2_Fe | 4.2 |
|  | CuSO_4_.5 H_2_O | 0.13 |
|  | ZnSO_4_·7 H_2_O | 0.67 |
|  | H_3_BO_3_ | 7 |
|  | MnSO_4_·H_2_O | 1 |
|  | (NH_4_)_6_Mo_7_O_24_.4 H_2_O | 0.33 |

**Table S9** Primer sequences used in promoter cloning and vector construction.

| Gene name | Primer sequence |
| --- | --- |
| GSP1 | 5′- GCAGAAGCCACCAGGGTCACGATTGTCAC-3′ |
| GSP2 | 5′- AGATAGCAGGAGGACACGGCGGCGATT-3′ |
| V5PF1 | 5′- CGCGGATCCACGACGAGAGAGACGTAGAAGA-3′ |
| V5PF2 | 5′- CGCGGATCCTCAACTCACCTCCCAAACGAAT-3′ |
| V5PF3 | 5′- CGCGGATCCAAGAATTGTTCAGCCTCACATTT-3′ |
| V5PR | 5′- CGGGGTACCGGTTTGAGACTTTTTGGAGTGTTTGG-3′ |
| V5F | 5′- CACCATGGTGGTTCCAAACCCATCTTCA-3′ |
| V5R | 5′- CATTATGAGTTCAGTGGGATAGG-3′ |

**Table S10** Primer information used in qRT-PCR detection.

| **ID** | **Gene name** | **Forward/Reverse** | **Primer sequence (5' to 3')** |
| --- | --- | --- | --- |
| AT4G25490 | CBF1 | Forward | GGAGACAATGTTTGGGATGC |
|  |  | Reverse | TTAGTAACTCCAAAGCGACACG |
| AT4G25470 | CBF2 | Forward | GACGTGTCCTTATGGAGCTATTAAAA |
|  |  | Reverse | TTACCATTTACATTCGTTTCTCACAAC |
| AT4G25480 | CBF3 | Forward | TTCCGTCCGTACAGTGGAAT |
|  |  | Reverse | AACTCCATAACGATACGTCGTC |
| AT1G27730 | ZAT10 | Forward | TCGAGCACTGGACAAAGGGTAAGC |
|  |  | Reverse | CCTCAGTGAGGTTTTGGTGGTGGA |
| AT5G59820 | ZAT12 | Forward | GTGCGAGTCACAAGAAGCCTAACA |
|  |  | Reverse | GCGACGACGTTTTCACCTTCTTCA |
| AT1G20440 | COR47 | Forward | CAGTGTCGGAGAGTGTGGTG |
|  |  | Reverse | ACAGCTGGTGAATCCTCTGC |
| At2G40140 | CZF1 | Forward | GCCTTGTCCCGAGTTTCGTA |
|  |  | Reverse | TGCGCGTACTCACACGAATC |
| At4G29190 | ZF | Forward | TCATTTCCTCGTAACAATCCTTTATTC |
|  |  | Reverse | CGGTGTTGTAGGCAGAGACTGA |
| AT1G07890 | APX1 | Forward | TCGCATGGCACTCTGCTGGAAC |
|  |  | Reverse | CACCAGTAACTTCAACGGCCAC |
| AT5G01600 | FER1 | Forward | CACTACTCCCTCACGGCTCTGCTT |
|  |  | Reverse | CGTTGTATTCCACATTGATTTGCTC |
| AT1G20630 | CAT1 | Forward | ACCTGTTGGTCGCTTGGTCTTGA |
|  |  | Reverse | GGTGAGCACATTTAGGGGCATTA |
| At1G62660 | ATVIN1 | Forward | AGTCCAGTGGCCAGTTGAGGA |
|  |  | Reverse | GCTGTGGAGCCTCCGTTTGT |
| At5G19510 | AtEF | Forward | GCTGTTCGTGGTGTTGAGATGC |
|  |  | Reverse | AGGCTCTGAGGTGAGGAAGTCT |
| KP053402 | *CsINV5* | Forward | AGTCTTGCCCCTTGATGTCG |
|  |  | Reverse | AACCAAACGGTCCAAGAGCA |
| GAAC01052498.1 | *CsPTB* | Forward | TGACCAAGCACACTCCACACTATCG |
|  |  | Reverse | TGCCCCCTTATCATCATCCACAA |
